# The insulin gene in humans and mice: evolution, regulation, and sex-specific mechanisms of diabetes susceptibility

**DOI:** 10.3389/fendo.2026.1954427

**Published:** 2026-10-20

**Authors:** Ali Mazloum, Nikita Batashkov, Veronika Ledyaeva, Gava Khulkhachiev, Dmitry Tabakov, Olga N. Mityaeva, Pavel Yu Volchkov

**Affiliations:** 1 Moscow Center for Advanced Studies, Moscow, Russia; 2 Federal Research Center for Innovator and Emerging Biomedical and Pharmaceutical Technologies, Moscow, Russia; 3 Moscow Clinical Scientific Center named after Anatoly Sergeevich (N. A. A. S.) Loginov, Moscow, Russia; 4 Faculty of Fundamental Medicine, Lomonosov Moscow State University, Moscow, Russia

**Keywords:** beta-cell biology, comparative genomics, diabetes mellitus, insulin gene (INS), insulin regulation, mouse models, precision medicine, sex differences

## Abstract

The insulin gene (INS) encodes a peptide hormone critical for glucose homeostasis, and its dysregulation underlies both type 1 and type 2 diabetes mellitus. This review compares the structural organization and functional regulation of the insulin gene in humans and mice, highlighting key species differences including the presence of two non-allelic insulin genes (Ins1 and Ins2) in mice versus a single INS gene in humans. Despite these differences, most preclinical diabetes research has historically been conducted in male mice, potentially concealing sex and species specific mechanisms. Additionally, this review examines the growing evidence for sex-specific differences in diabetes susceptibility. Recent single-cell multi-omics studies reveal that human pancreatic islets exhibit sex-specific chromatin accessibility and gene expression patterns, with autosomal sex differences emerging predominantly in type 2 diabetes. The protective effects of estrogen, mediated through estrogen receptor α signaling, contribute to enhanced insulin sensitivity and beta-cell function in premenopausal females. We argue that routine inclusion of both sexes and species aware interpretation of insulin gene studies are prerequisites for precision diabetology.

## 1 Introduction

Diabetes mellitus (DM) is a chronic metabolic disorder characterized by persistent hyperglycemia resulting from defects in insulin secretion and/or insulin inefficiency (1). According to the International Diabetes Federation (IDF) Atlas 11th edition, DM represents one of the fastest-growing global health challenges of the 21st century (2). An estimated 589 million adults aged 20–79 worldwide are living with diabetes, with projections exceeding 852.5 million by 2050 (45% increase).

Several types/subtypes of the disease have been identified, which differ in etiology, pathophysiology, and management. The most prevalent being type 2 diabetes (T2D), accounting for 90–95% of all cases. Type 1 diabetes (T1D) contributes to between 5% and 10%, while other rare forms account for 1–3% of the global cases.

Type 1 diabetes is characterized as an autoimmune condition wherein the immune system selectively targets and destroys pancreatic β-cells, culminating in absolute insulin deficiency (3). Although its onset most commonly occurs during childhood or adolescence, it may also emerge in adulthood, like “latent autoimmune diabetes of adulthood” (LADA) which is slowly progressive and often misdiagnosed as T2D, necessitating lifelong exogenous insulin therapy alongside careful attention to diet and lifestyle (4). Type 2 diabetes (T2D), by contrast, arises from the interplay of peripheral insulin resistance and progressive β-cell dysfunction; while traditionally linked to obesity and lifestyle factors in middle-aged adults, its prevalence is rising among younger populations due to increasing rates of sedentary behavior and obesity (5). Beyond these two predominant forms, monogenic diabetes syndromes, such as Maturity Onset Diabetes of the Young (MODY), represent a distinct subset of diabetes that frequently remains misdiagnosed in clinical practice. MODY subtypes are caused by one or more mutations in a single gene, often in transcription factors (HNF1A and HNF4A), Insulin gene (INS), or other genes involved in insulin synthesis. To date, 14 subtypes of MODY have been identified, based on the gene affected (6).

The insulin hormone, discovered nearly a century ago, remains central to our understanding of glucose metabolism and diabetes pathogenesis. Insulin is synthesized exclusively in pancreatic beta cells, where it is produced, stored, and released in response to elevated blood glucose concentrations. The gene encoding insulin has been extensively studied across species, revealing both conserved features and intriguing evolutionary differences (7).

Although insulin biology has been investigated for more than a century, important biological variables remain underappreciated in both experimental and clinical diabetes research. Most mechanistic studies continue to rely on murine models despite fundamental differences between humans, who possess a single INS gene, and mice, which express two non-identical insulin genes, Ins1 and Ins2, with distinct evolutionary origins, regulatory mechanisms, and immunological functions. At the same time, growing evidence demonstrates that biological sex significantly influences β-cell physiology, insulin secretion, glucose homeostasis, immune regulation, and susceptibility to both type 1 and type 2 diabetes. These two dimensions (species-specific insulin gene biology and sex-dependent metabolic regulation) are often considered independently, despite their combined impact on the interpretation and translation of experimental findings. By integrating these complementary fields, this review provides a unified perspective on how evolutionary divergence of insulin genes and biological sex jointly influence diabetes susceptibility, interpretation of experimental models, and the translation of preclinical findings into human disease. We further discuss how recognition of these factors may improve future experimental design and support the development of more precise therapeutic strategies.

## 2 Literature search and selection strategy

This review was conducted as a comprehensive narrative synthesis of literature addressing insulin gene evolution and regulation, comparative human and murine insulin biology, experimental models of diabetes, and sex-dependent mechanisms of β-cell dysfunction. Relevant literature was identified through searches of PubMed/MEDLINE, Web of Science, and Google Scholar using combinations of the terms ‘INS’, ‘Ins1’, ‘Ins2’, ‘insulin gene’, ‘β-cell’, ‘pancreatic islet’, ‘diabetes’, ‘sex differences’, ‘sex hormones’, ‘sex chromosomes’, ‘single-cell RNA sequencing’, ‘single-nucleus ATAC sequencing’, ‘multi-omics’, and specific experimental models including ‘STZ’, ‘NOD’, ‘Akita’, ‘ob/ob’, ‘db/db’, and ‘ZFDM’. Priority was given to primary research articles, major datasets, and recent studies providing mechanistic, transcriptomic, genomic, or clinical evidence, while landmark earlier studies were retained where they established foundational concepts or experimental models. For clinical therapeutic comparisons, preference was given to randomized clinical trials, cardiovascular and renal outcome trials, and systematic reviews or meta-analyses. For human islet transcriptomic and single-cell analyses, studies were selected when sex-stratified analyses and relevant donor-level information were available. Because this article is a narrative review rather than a systematic review or meta-analysis, no formal risk-of-bias scoring or quantitative pooling was performed. The evidence was therefore synthesized with attention to species, model, experimental, and donor-level limitations.

## 3 The insulin gene: structure, biosynthesis and function

Although insulin performs a highly conserved physiological function across mammals, the genetic organization and regulation of insulin differ considerably between humans and mice. These differences are not merely evolutionary curiosities; they influence insulin biosynthesis, immune tolerance, and the interpretation of experimental models used to investigate diabetes. Understanding how the single human INS gene compares with the duplicated murine Ins1 and Ins2 genes therefore provides the foundation for interpreting species-specific mechanisms of diabetes susceptibility. This section examines the evolutionary origin, genomic organization, and regulatory characteristics of insulin genes in humans and mice, establishing the molecular framework for the translational differences discussed in subsequent sections.

### 3.1 Human insulin gene

The human insulin gene (INS) resides on the short arm of chromosome 11 (11p15.5) and spans approximately 1,430 base pairs. It contains two introns and three exons, a structural organization that is highly conserved across mammals and serves as the evolutionary reference for comparative analyses with murine insulin genes. Exon 1 encodes the 5′ untranslated region and part of the preproinsulin signal peptide, whereas exons 2 and 3 encode the remainder of the preproinsulin sequence, including the B-chain, C-peptide, and A-chain. Unlike mice, humans possess only a single insulin gene, making INS the sole source of endogenous insulin throughout life and the evolutionary baseline for comparative studies of insulin gene biology.

Mature insulin synthesis begins with transcription of the INS gene, producing preproinsulin mRNA. This mRNA is translated by ribosomes on the rough endoplasmic reticulum (ER) into preproinsulin—a 110-amino-acid precursor composed of an N-terminal signal peptide (24 aa), the B chain (30 aa), the C-peptide (35 aa), and the A chain (21 aa) (**Figures 1**, **2**, steps 1 and 2).

**Figure 1:**
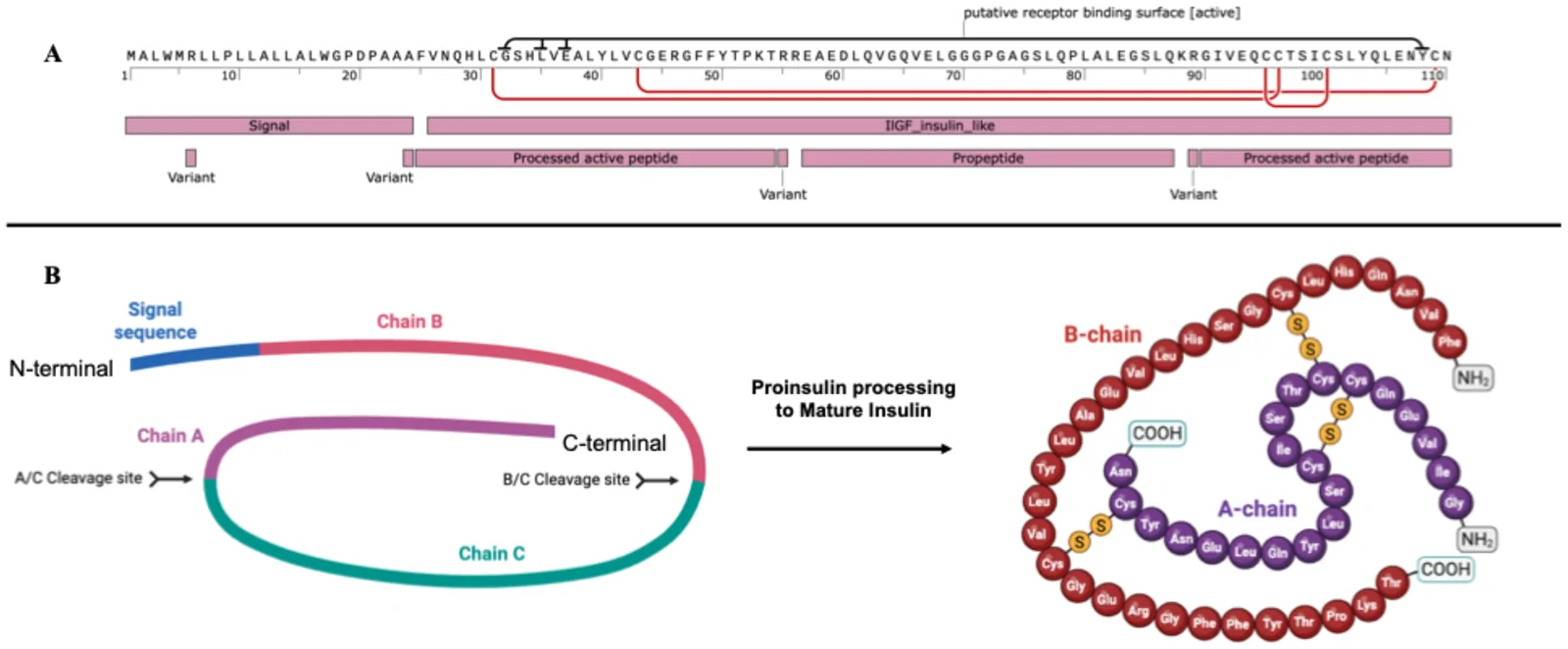
Structure of proinsulin and mature insulin. **(A)** The full coding amino acid sequence of human insulin showing all its features; **(B)** Structure of proinsulin and mature insulin showing the peptide chains, two disulfide bonds between the B-chain and the A-chain, and one disulfide bond of the A-chain. Reproduced from Mazloum et al. (6) under the Creative Commons Attribution 4.0 International License (CC BY 4.0).

**Figure 2:**
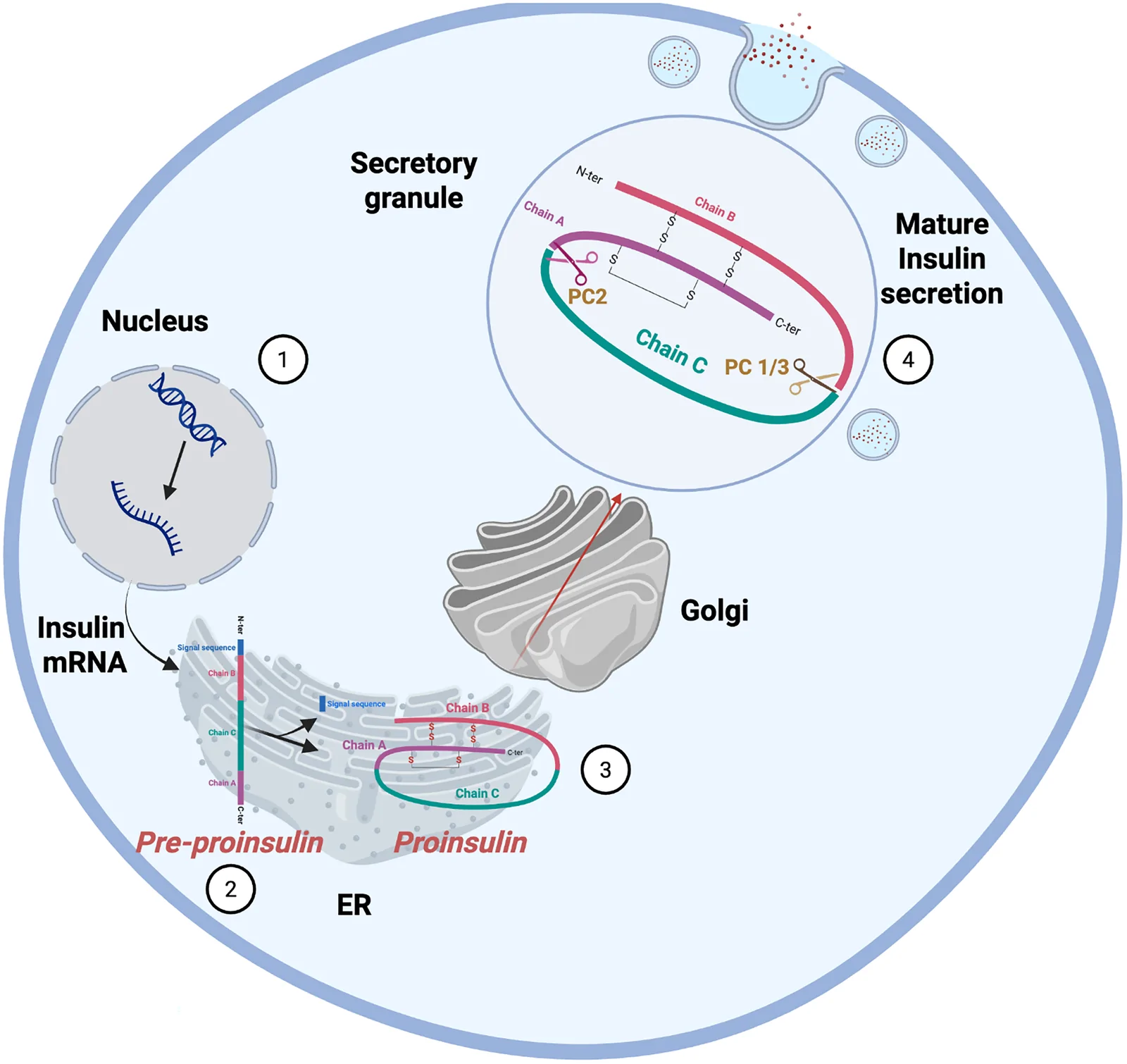
Pathway of insulin biosynthesis from transcription to mature insulin secretion. Reproduced from Mazloum et al. (6) under the Creative Commons Attribution 4.0 International License (CC BY 4.0).

Once preproinsulin enters the ER lumen, its signal peptide is cleaved, yielding proinsulin (86 amino acids). Proinsulin then folds properly with the assistance of chaperones such as BiP and PDIA1 (Protein Disulfide Isomerase A1), during which three critical disulfide bonds form (A6–A11, A7–B7, and A20–B19) to stabilise the structure (**Figure 2**, steps 2 and 3).

Correctly folded proinsulin is transported to the Golgi apparatus via COPII-coated vesicles. Within the Golgi, proinsulin is sorted, concentrated, and packaged into clathrin-coated immature secretory granules that bud from the trans-Golgi network. Inside these granules, proinsulin is cleaved by prohormone convertases: PC1/3 at Arg31-Arg32 (separating the B chain from the C-peptide) and PC2 at Lys64-Arg65 (separating the C-peptide from the A chain). Carboxypeptidase E subsequently removes the exposed basic residues, producing mature insulin (51 amino acids, consisting of the A and B chains linked by disulfide bonds) and the C-peptide. Both mature insulin and C-peptide are secreted in equimolar amounts (**Figure 2**, step 4). Mature insulin is stored in secretory granules together with Zn²^+^ crystals; upon glucose stimulation, Ca²^+^-dependent exocytosis releases insulin into the bloodstream to regulate glucose uptake. Meanwhile, the Golgi apparatus also glycosylates other secretory proteins, sorts cellular cargo, synthesizes lipids such as sphingomyelin, and maintains membrane integrity for granule formation (6).

The human INS gene is among the most evolutionarily conserved genes involved in endocrine regulation, reflecting the essential role of insulin in vertebrate glucose homeostasis. Despite strong conservation of the coding sequence, regulatory regions have evolved in a species-specific manner, contributing to differences in tissue-specific expression, developmental regulation, and disease susceptibility. These regulatory differences become particularly important when comparing human insulin biology with that of rodents, which possess two insulin genes with partially specialized functions.

Transcription of the INS gene is highly tissue-specific, being almost exclusively restricted to pancreatic beta cells. This specificity is achieved through a complex promoter region containing multiple regulatory elements that bind transcription factors including PDX1, NeuroD1, and MafA (8; Y. 9). In addition to these core transcription factors, chromatin organization, epigenetic modifications, and long-range regulatory elements contribute to the precise spatial and temporal control of INS expression. Among these, the variable number tandem repeat (VNTR) located upstream of the INS promoter has attracted considerable attention because of its association with thymic insulin expression and susceptibility to type 1 diabetes (8). Although the functional consequences of the VNTR are discussed in later sections, its presence indicates that regulation of the human INS locus extends beyond the coding sequence.

### 3.2 Mouse insulin genes: a unique duplication event

Mouse insulin goes through the same fundamental mechanism of insulin biosynthesis: translating preproinsulin, cleaving it to proinsulin, and then to mature insulin just like the one described earlier for human INS. However, they still have several key differences in proinsulin structure, gene expression and regulation, processing location and glucose sensing mechanism, but the most significant one is that mice possess two non-allelic insulin genes (Ins1 and Ins2).

Shiao et al. (10) focused on the rodent two-gene system, demonstrating that Ins1 originated as a retroposed copy of Ins2 approximately 20 million years ago in the common ancestor of Murinae (10–12). In his study, using Southern blot analyses and PCR sequencing across nine murid species confirmed that Ins1 is present only in murine rodents (subfamily Murinae), while more distantly related species like hamsters and voles carry only a single copy (Ins2), establishing Ins1 as a relatively young, lineage-specific retrogene (13, 14). Despite being a young retrogene, Ins1 is maintained under strong purifying selection across all functional domains, including the C-peptide (15). Unexpectedly, population genetic analyses in *Mus musculus domesticus* revealed signatures of recent positive selection on both Ins1 and Ins2, with an excess of rare alleles and three specific amino acid sites (two in the signal peptide, one in the C-peptide) evolving under positive selection (16–18). The authors proposed an extension of the thrifty-genotype hypothesis, suggesting that Ins1 may have conferred metabolic advantages under ancestral resource-scarce conditions but became a diabetes risk factor after environmental shifts associated with agriculture, prompting adaptive evolution in both genes to mitigate disease susceptibility (19, 20).

Irwin (7) placed these findings within a broader vertebrate perspective, showing that lineage-specific insulin gene duplications are widespread and have arisen through multiple mechanisms—retroposition (as in rodents and some fish), whole-genome duplication (as in teleosts and Xenopus), and local duplications (**Figure 3**) (11, 12, 21–24). Many duplicated insulin genes exhibit divergent expression patterns and, in some cases, altered proteolytic processing sites that prevent cleavage into the classic two-chain hormone, instead producing single-chain insulin-like molecules that retain conserved cysteine residues and may have distinct functions (22, 25, 26). This challenges the long-held view that two-chain processing is obligatory for biological activity and aligns with the observation in rodents that both Ins1 and Ins2 remain functionally constrained despite their different evolutionary histories (27, 28). Together, the two articles illustrate that insulin gene evolution is characterized by recurrent duplication, adaptive fine-tuning under shifting selective pressures, and surprising structural plasticity—all of which contribute to the diversity of insulin function across vertebrates and offer insights into the etiology of diabetes (29–31).

**Figure 3:**
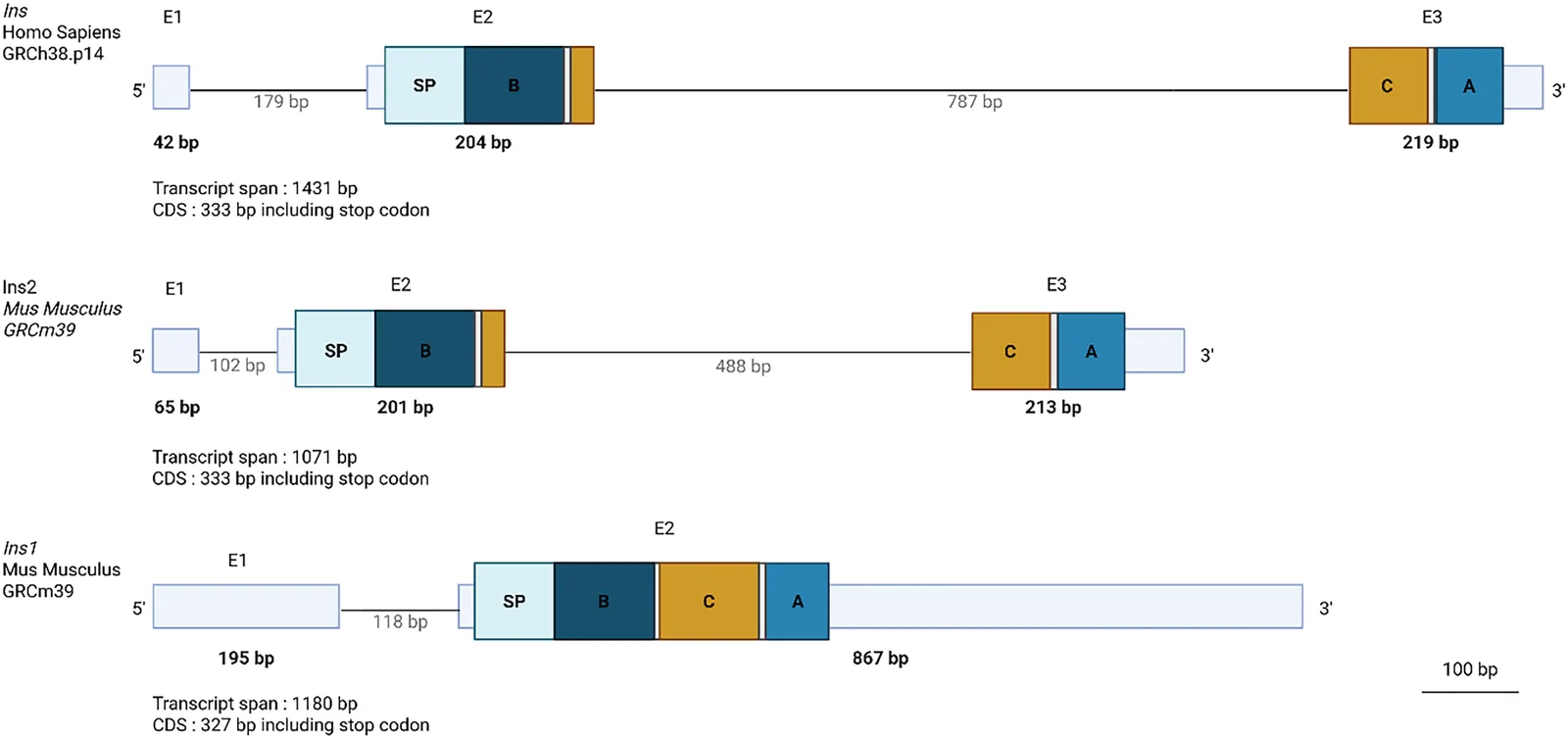
Exon–intron organization and encoded peptide regions of human *INS* and mouse *Ins2* and *Ins1*. Regions encoding the signal peptide (SP), B chain, C-peptide and A chain are shown in light cyan, dark blue, ochre and blue, respectively. Grey bars indicate the dibasic proinsulin processing sites between the B chain and C-peptide and between C-peptide and the A chain. Untranslated regions are shown as pale blue boxes, and introns as connecting lines. Exon and intron lengths are indicated in base pairs (bp). Note that according to RefSeq (NM_008386.4), mouse *Ins1* exon 2 is shorter than the Ensembl annotation shown here (406 versus 867 bp), reflecting a shorter annotated 3′ untranslated region.

These findings indicate that the murine insulin gene system represents an evolutionary innovation that increases biological complexity rather than simple gene redundancy. Consequently, differences between human and murine insulin genes should be interpreted as functionally meaningful adaptations rather than species-specific curiosities.

### 3.3 Pancreatic islet organization and functional microenvironment

Although insulin gene regulation is governed by highly conserved molecular mechanisms, this hormone is produced in the beta-cells of the pancreas whose cellular organization differs substantially between mice and humans. Because islet architecture influences cell-cell communication, vascularization, innervation, and paracrine signaling, these structural differences contribute to species-specific regulation of β-cell function and should be considered when interpreting experimental findings.

Insulin plays a vital regulatory role in metabolism. This hormone increases the storage of glucose, fatty acids, and amino acids by its action on the liver, adipose tissue and muscle. In humans, the endocrine pancreas, also known as the islets of Langerhans, is composed of discrete cells, dispersed throughout the mass of the whole pancreas. On the contrary, the islets of mice are centered in the whole mass of the pancreas, surrounded by four other endocrine cell types, alpha, beta, epsilon and pancreatic polypeptide (PP) (32). These four cells are responsible for the synthesis of hormones like glucagon, somatostatin, ghrelin and PP, respectively.

In recent years, human islet structure and cellular organization have been more carefully scrutinized, leading to new questions and controversies regarding the differences between islets in man and mouse. Although there is consensus on the cell types seen within human and rodent islets, a lower proportion of beta cells are found in humans compared with rodents. There is also ongoing debate on topographic endocrine cell arrangement within human islets. While there are also many similarities, the composition, architecture, innervation, and function of human islets are quite different from those seen in mice (33–36). Human beta cells often are not the only cell clustered in the center of an islet as they are in rodents. In contrast to mice, some studies have demonstrated that more than 70% of human beta cells have direct physical associations with other endocrine cells (e.g., alpha, delta, and gamma/pancreatic polypeptide cells), suggesting that unique paracrine interactions may occur between beta cells and their immediate neighbors (34). Other studies have reported heterogeneity among human islets, including different architecture within islets depending on their size (**Figure 4**) (37).

**Figure 4:**
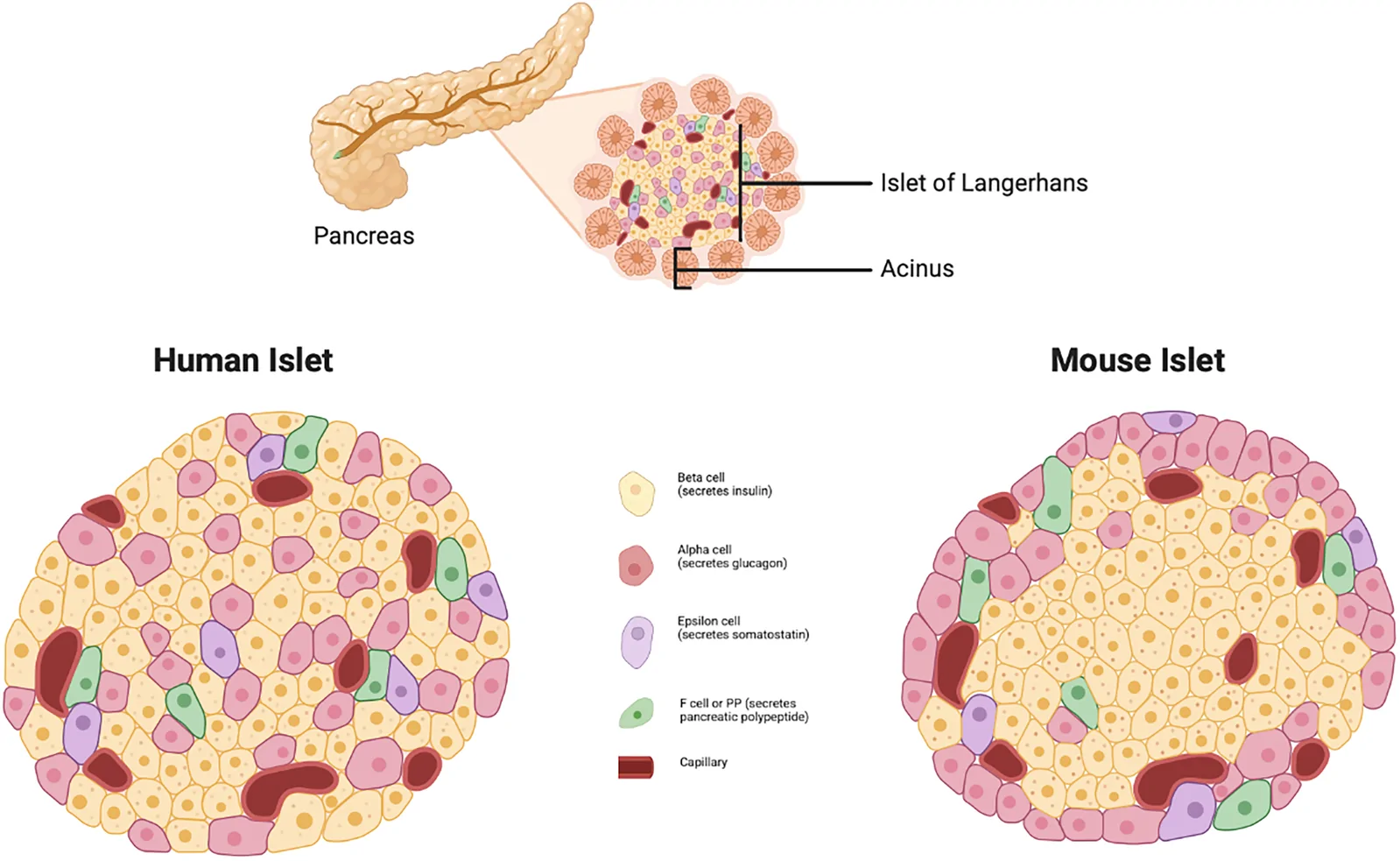
Architecture of mouse and human islets. The above images demonstrate differences between islets in men and mice.

These anatomical differences indicate that insulin secretion occurs within distinct cellular microenvironments in humans and mice. Consequently, β-cell function should be interpreted within the context of species-specific islet architecture, providing important background for understanding differences in insulin gene regulation.

### 3.4 Molecular regulation of insulin gene expression

Despite differences in islet architecture, insulin gene transcription is regulated by a highly conserved network of transcription factors that establishes β-cell identity and couples insulin production to metabolic demand. While this core regulatory machinery is largely conserved across mammals, important differences exist between the human INS promoter and the murine Ins1 and Ins2 promoters, reflecting their distinct evolutionary histories.

Insulin gene transcription is primarily regulated by glucose concentration, with additional modulation by hormones, nutrients, and neural inputs. Glucose stimulation increases proinsulin mRNA levels, indicating that transcriptional regulation contributes significantly to glucose-stimulated insulin biosynthesis.

Insulin transcription is controlled by a hierarchical network of transcription factors that establish β-cell identity while dynamically responding to metabolic stimuli. These transcription factors bind to multiple conserved regulatory elements of the insulin gene promoter (**Figure 5**). The A-box elements bind PDX1 (pancreatic and duodenal homeobox 1), a master regulator of beta-cell development and function. E-box elements bind NeuroD1 and other basic helix-loop-helix factors, while C-box elements bind MafA, a key regulator of glucose-responsive insulin transcription. Mutations affecting these regulatory elements or their cognate transcription factors can impair insulin expression and contribute to diabetes pathogenesis.

**Figure 5:**
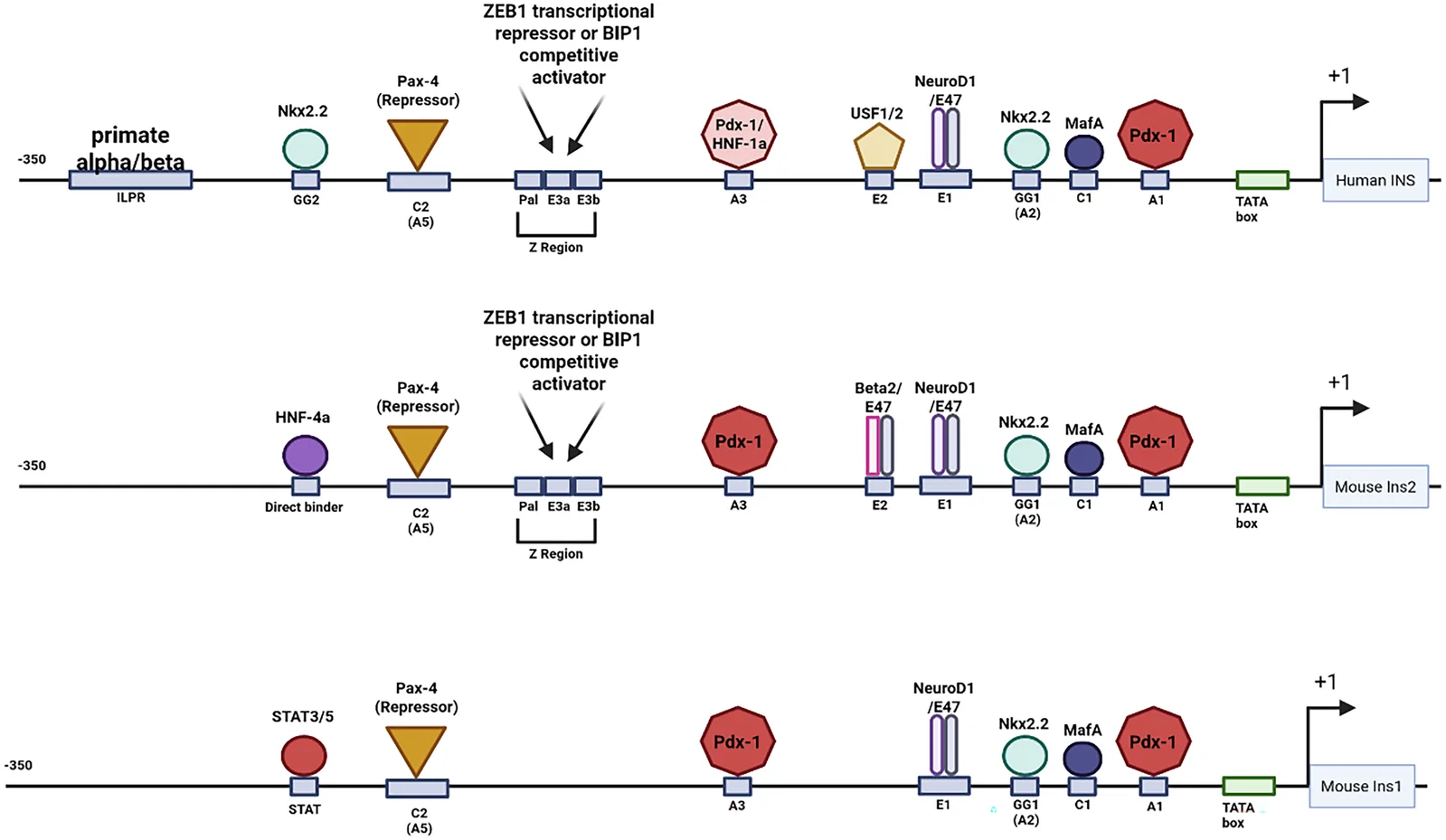
The 5’ enhancer of insulin gene in the human genome in comparison to the two insulin genes of mice.

The insulin enhancer is primarily activated through a conserved “core cassette” of A, C, and E elements, with cooperative interactions among transcription factors, coactivators, and the basal transcriptional machinery driving cell-specific and glucose-regulated expression (28). Functional synergy among MafA, PDX-1, and BETA2/NeuroD1 is required for high-level expression; while individual factors have weak effects, they physically interact to produce strong synergistic activation. MafA physically interacts with PDX-1 and BETA2, and dominant-negative MafA or siRNA drastically reduces insulin transcription in beta cells (38). PDX-1 (IPF1/STF-1) binds to the A3 site and is essential for beta-cell development and function, interacting with p300/CBP coactivators to recruit the transcriptional machinery (39, 40). MafA (RIPE3b1) is restricted to insulin-producing cells and mediates glucose-regulated and fatty-acid-inhibited expression (41, 42), while the BETA2/NeuroD1 heterodimer binds the E-element (CANNTG), a role so critical that its absence leads to severe diabetes due to beta-cell loss (41, 43, 44).

Regulation is further modulated by the C2 element, where the activator PAX6 (45) is frequently opposed by the related PAX4, which represses PAX6-mediated transactivation to maintain cellular identity (46, 47). Beyond this core machinery, significant divergence exists between species. The ancestral human *INS* and mouse *Ins*2 genes share a complex distal architecture, including the Z control region (–292/–243 bp), which contributes to glucose-inducible transcription and acts as a repressor in non-beta cell types; its activity depends partly on the recruitment of PDX-1 and MafA (48). In stark contrast, the rodent *Ins*1 promoter is a “stripped-down” retrocopy of *Ins*2. Because it originated from a processed mRNA retroposition, it lacks the distal Z-region and E2 elements found in the ancestral genes. However, *Ins*1 has evolved unique compensatory features, such as a STAT element (binding STAT3/5) that allows it to respond to hormonal cues like prolactin. Finally, species-specific markers define these promoters: rodents possess a direct binding site for HNF-4α, whereas the human promoter features the unique ILPR (Insulin-Linked Polymorphic Region)—a series of tandem repeats that regulates chromatin accessibility and serves as a major genetic susceptibility locus for Type 1 Diabetes.

This demonstrates that insulin gene expression is controlled by a conserved transcriptional framework that has undergone species-specific evolutionary adaptation. These regulatory differences provide the mechanistic basis for divergent insulin biology in humans and mice and influence the interpretation of experimental diabetes models.

### 3.5 Comparative genomic context

Beyond the coding sequence and promoter architecture of the insulin gene, its broader genomic context provides important insights into its evolutionary history and regulatory organization. Comparative genomic analyses have shown that genes rarely evolve in complete isolation; instead, they are often embedded within conserved chromosomal neighborhoods that preserve regulatory interactions across millions of years of evolution. Examining the genomic environment surrounding the insulin locus therefore offers an additional perspective on the evolutionary forces that have shaped insulin biology in vertebrates.

The genomic region encompassing the insulin gene is embedded within an evolutionarily conserved chromosomal neighborhood that includes the flanking genes tyrosine hydroxylase (TH) and insulin-like growth factor 2 (IGF2). In humans, these genes are arranged in close proximity on chromosome 11p15, forming the canonical TH–INS–IGF2 locus. Early molecular mapping studies demonstrated that INS and IGF2 are contiguous, with TH positioned immediately upstream, establishing the structural organization of this locus (49). Remarkably, the genomic neighborhood surrounding insulin genes shows extensive synteny across evolutionarily distant species. The flanking genes tyrosine hydroxylase (TH) and insulin-like growth factor 2 (IGF2) are preserved upstream and downstream of the insulin locus in species ranging from zebrafish to humans, reflecting over 450 million years of evolutionary conservation. This synteny suggests that the chromosomal region containing insulin has been maintained due to critical regulatory interactions, though the functional significance of this conservation remains incompletely understood. Analyses of the IGF2 locus in nonmammalian vertebrates have shown that neighboring genes, including TH, are retained in a conserved configuration, supporting the concept of an ancient genomic block (50). Importantly, the conservation is not absolute. In certain teleost species, such as barramundi, TH and IGF2 remain adjacent while INS is absent from the same linkage group, likely reflecting the teleost-specific whole genome duplication and subsequent gene loss. This indicates that the ancestral locus can be partially disrupted without complete loss of syntenic identity (51). These observations suggest that the conserved unit is better understood as a regulatory genomic neighborhood rather than a strictly invariant gene order.

The persistence of the TH–INS–IGF2 locus across vertebrates implies the presence of evolutionary constraints maintaining this genomic configuration. In mammals, this region is embedded within a complex regulatory landscape, particularly due to the presence of IGF2, which resides in an imprinted domain subject to long-range cis-regulatory control. At the same time, comparative studies indicate that the regulatory architecture of the locus has diverged across vertebrate lineages. In nonmammalian vertebrates, the Igf2 locus is structurally simpler, lacking the full imprinting-associated regulatory complexity observed in mammals (50). This suggests that while the locus is ancient, its regulatory features have evolved progressively.

Several lines of evidence suggest potential functional interactions between genes within the TH–INS–IGF2 locus. In addition to the possibility of shared cis-regulatory elements, such as beta-cell-specific enhancers that coordinate INS and IGF2 expression through chromatin looping, one of the most notable findings is the existence of chimeric transcripts spanning TH and INS. In avian systems, regulated expression of TH–INS fusion transcripts has been demonstrated during development, producing altered tyrosine hydroxylase isoforms with reduced enzymatic activity (52). These results indicate that physical proximity between genes can facilitate transcriptional read-through and generate novel functional transcripts.

Similar transcriptional complexity has been described in mammals. In marsupials, the TH/INS locus produces multiple transcript isoforms, including chimeric RNAs and long noncoding transcripts, suggesting that this genomic region can function as a transcriptionally integrated unit (53). These comparative genomic data reveal that the insulin locus is embedded within an evolutionarily conserved yet dynamically evolving transcriptional neighborhood, a complexity that has profound implications for interpreting the impact of non-coding variants on insulin regulation and susceptibility to metabolic disease.

The evidence presented in this section demonstrates that human and murine insulin genes differ not only in gene number but also in their evolutionary history, genomic organization, transcriptional regulation, and physiological roles. While the human INS gene represents a single, highly regulated locus, the murine Ins1 and Ins2 genes have undergone functional specialization that influences β-cell biology and immune tolerance. These fundamental differences highlight important limitations when extrapolating findings from mouse models to human diabetes and provide the molecular basis for understanding the species-specific disease mechanisms discussed in the following sections.

### 3.6 Intracellular Ca2+ homeostasis, ER stress, and proinsulin processing

Insulin biosynthesis places a substantial proteostatic and calcium-handling demand on pancreatic β-cells. After translation into the rough endoplasmic reticulum (ER), preproinsulin enters a Ca2+-dependent folding environment in which molecular chaperones and protein-disulfide isomerases support proinsulin folding and disulfide-bond formation. Correct folding is therefore coupled to ER Ca2+ homeostasis, protein quality control, trafficking from the ER to the Golgi, and subsequent packaging into secretory granules. When insulin biosynthetic demand exceeds ER folding capacity, activation of the unfolded protein response (UPR) can initially restore proteostasis but, if sustained, can suppress protein synthesis and activate apoptotic pathways (54). These processes provide a mechanistic bridge between insulin gene transcription, proinsulin biosynthesis, ER stress, and β-cell survival (55, 56).

Intracellular Ca2+ dynamics also connect the ER to mitochondria and to glucose-stimulated insulin secretion. Voltage-gated Ca2+ entry at the plasma membrane triggers the first phase of insulin granule exocytosis, while ER Ca2+ release and store-operated Ca2+ entry contribute to sustained secretion. Mitochondrial Ca2+ uptake links cytosolic Ca2+ signals to ATP production, thereby amplifying KATP-channel-dependent depolarization and insulin exocytosis. At mitochondria-associated ER membranes (MAMs), disruption of ER-to-mitochondria Ca2+ transfer can therefore simultaneously impair ATP generation, redox balance, and secretory function. This integrated ER–mitochondrial network is particularly relevant to diseases in which insulin misfolding and ER stress are primary drivers of β-cell dysfunction.

Wolfram syndrome provides an important genetic example of this relationship. WFS1 (wolframin) is an ER-resident protein involved in ER Ca2+ homeostasis and the unfolded protein response. Loss of WFS1 function causes ER Ca2+ depletion, abnormal cytosolic Ca2+ signaling, ER stress, and activation of cell-death pathways in β-cells. CISD2, the gene responsible for Wolfram syndrome type 2, is localized to the ER and mitochondria-associated membranes and contributes to Ca2+ homeostasis, mitochondrial function, proteostasis, and insulin granule maturation. Recent β-cell-specific Cisd2-deficiency studies demonstrated impaired glucose-induced Ca2+ influx, mitochondrial dysfunction, abnormal β-granule maturation, and reduced insulin secretion. Thus, WFS1/CISD2-associated diabetes illustrates how calcium homeostasis, proinsulin processing, vesicle trafficking, and β-cell survival are mechanistically interconnected (56, 57).

These mechanisms are also directly relevant to insulin-gene regulation. Increased glucose and secretory demand stimulate INS/Ins transcription, but persistent ER stress can feed back on transcriptional and translational programs through UPR signaling, altering β-cell identity and reducing the capacity to synthesize, fold, and secrete insulin. Consequently, insulin gene expression should not be considered in isolation from the intracellular environment in which proinsulin is folded and processed. Species-specific differences in Ins1/Ins2 organization and β-cell physiology may further modify how these stress responses are manifested in mouse models compared with the single human INS system.

## 4 Species-specific implications of insulin gene biology in diabetes

The molecular and evolutionary differences between human and murine insulin genes extend beyond comparative genomics and have important functional consequences for diabetes susceptibility and disease progression. Variations in gene number, regulatory architecture, and tissue-specific expression influence β-cell physiology, immune tolerance, and the response to metabolic stress, thereby shaping the pathogenesis of both type 1 and type 2 diabetes. Consequently, understanding these species-specific mechanisms is essential for interpreting findings derived from experimental mouse models and for assessing their translational relevance to human disease. This section examines how differences in insulin gene biology contribute to diabetes pathogenesis, highlighting both the strengths and limitations of murine models in diabetes research.

### 4.1 Implications of the two-gene system

The presence of two insulin genes, Ins1 and Ins2, in mice has important implications for diabetes research Ins1 and Ins2, which encode highly homologous proinsulin proteins but differ significantly in their immunological impact. Ins2, the ancestral gene, is expressed both in pancreatic β-cells and in the thymus, where it contributes to central tolerance through presentation via MHC class II molecules on medullary thymic epithelial cells. In contrast, Ins1 expression is largely restricted to pancreatic β-cells and is minimal or absent in the thymus (58). This differential expression has profound consequences: thymic Ins2 expression promotes deletion of high-affinity insulin-reactive T cells, whereas Ins1-specific T-cell clones can escape negative selection (58). Studies using knockout mice have revealed that Ins1 and Ins2 are not functionally redundant. Mice lacking Ins2 develop mild glucose intolerance and beta-cell hyperplasia, while combined Ins1/Ins2 deficiency results in severe diabetes with ketoacidosis. Ins1 knockout alone produces relatively mild phenotypes, suggesting that Ins2 may play a more critical role in maintaining glucose homeostasis.

Central tolerance to insulin is largely mediated by thymic expression of Ins2. In the thymus, proinsulin-derived peptides are presented by MHC class II molecules on medullary thymic epithelial cells (mTECs), leading to deletion of high-affinity autoreactive CD4^+^ T cells. This process is critical for establishing tolerance to insulin as a self-antigen. Because Ins1 is minimally expressed in the thymus, T-cell clones specific for Ins1-derived epitopes-particularly InsB:9–23-may escape negative selection. As a result, the peripheral T-cell repertoire retains potentially pathogenic clones capable of recognizing insulin in pancreatic islets.

Genetic studies further support this model. Deletion of Ins1 protects NOD mice from diabetes, whereas deletion of Ins2 accelerates disease onset, indicating a tolerogenic role for Ins2 and a dominant autoantigenic role for Ins1 (59). At the mechanistic level, the presentation of the B:9–23 epitope by the NOD-specific MHC class II molecule I-Ag7 is critical. I-Ag7 lacks Asp at position β57, resulting in an altered peptide-binding groove that accommodates peptides with relatively low stability and permits binding in multiple registers. Notably, distinct binding registers of the B:9–23 peptide differ in their immunogenicity; certain registers important to autoimmunity reaction are poorly represented during thymic selection but efficiently stimulate autoreactive CD4^+^ T cells in the periphery (60).

Despite the high sequence similarity between Ins1- and Ins2-derived proinsulin, even minor amino acid differences can influence antigen processing and MHC loading, thereby shaping the repertoire of presented epitopes. In addition, β-cells can generate unconventional peptide species, including alternative cleavage products and hybrid insulin peptides, which are not encountered during thymic education and may further contribute to the breakdown of tolerance (61). Collectively, these findings indicate that the immunodominance of Ins1 is not primarily driven by gross structural differences in the encoded protein, but rather by differential thymic expression and epitope presentation dynamics, which together define the autoreactive T-cell repertoire and susceptibility to autoimmune diabetes.

Taken together, current evidence supports a model in which the immunodominance of Ins1 arises from a combination of factors. The absence of significant thymic expression prevents effective central tolerance to Ins1-derived epitopes. Meanwhile, structural features of I-Ag7 enable unconventional presentation of key epitopes such as InsB:9–23, including pathogenic binding registers that evade thymic selection. Finally, β-cell–specific antigen processing generates peptide variants that further expand the pool of immunogenic epitopes.

Thus, despite the high sequence similarity between Ins1 and Ins2, their distinct expression patterns and differences in antigen presentation dynamics lead to fundamentally different immunological outcomes. Ins2 functions primarily as a tolerogenic antigen, whereas Ins1 serves as the dominant trigger of autoreactive T-cell responses in autoimmune diabetes.

The existence of two insulin genes in mice provides a valuable experimental system for studying gene dosage effects and regulatory evolution. However, researchers must exercise caution when extrapolating findings from mouse models to human disease, as the two-gene system may introduce compensatory mechanisms not present in humans.

### 4.2 Functional consequences of regulatory divergence

Beyond gene copy number, regulatory mechanisms controlling insulin expression may differ between species. The human INS gene contains a polymorphic variable number tandem repeat (VNTR) region upstream of the transcription start site, which has been associated with diabetes susceptibility. Class I VNTR alleles (shorter repeats) are associated with increased risk of T1D, while class III alleles (longer repeats) may influence T2D risk. Similar regulatory polymorphisms have been identified in mouse Ins genes, though their functional significance may differ.

The INS gene contains a VNTR in the form of a 14-bp repeating unit, in its 5’ control region. These VNTRs can be divided into three classes according to the number of repeating units present. Lucassen et al. (62) first described an association of the length of this VNTR with susceptibility to insulin-dependent diabetes mellitus (IDDM) (62). Subsequently, two independent researchers conducted by 63 and 64 conducted that the INS VNTR influences the expression of insulin transcript in fetal thymus: INS alleles with a class III VNTR (140–210 repeats) express the insulin more abundantly than those with a class I VNTRs (26–63 repeats) (63, 64). The higher level of insulin expression in the thymus is considered to promote negative selection of insulin-specific T-lymphocytes, and thereby play a critical role in the pathogenesis of IDDM. Furthermore, Bennett et al. (65) found that the insulin VNTR allele-specific effect in IDDM depended on the identity of the untranslated paternal allele (65, 66).

The main finding of Durinovic-Bello and colleagues is that the insulin gene VNTR genotype directly determines the frequency of peripheral CD4+ T cells reactive to the proinsulin epitope PI(76–90). Using HLA-DRB1*0401 tetramers, they showed that individuals with the diabetes-susceptible INS-VNTR class I/I genotype had readily detectable proinsulin-specific T cells (79% of subjects), whereas those carrying at least one protective class III allele (III/x) had markedly lower frequencies (29% of subjects; P < 0.001). This association was specific to proinsulin and independent of disease status (type 1 diabetes, autoantibody-positive at-risk individuals, or healthy controls). When a modified “9S” agonist tetramer that stabilizes low-avidity T-cell binding was used, however, both genotype groups showed similar levels of proinsulin-reactive T cells. This indicates that the protective VNTR III haplotype does not eliminate all proinsulin-specific T cells but rather selectively deletes high-avidity autoreactive clones, likely through higher thymic insulin expression and more efficient central tolerance. Thus, the INS-VNTR genotype controls diabetes susceptibility by setting the avidity threshold of the autoreactive T-cell repertoire to proinsulin (8).

### 4.3 Common mouse models of diabetes and their relevance to insulin gene studies

Murine models are central to the study of diabetes mellitus, enabling controlled investigation of disease mechanisms and preclinical evaluation of therapeutic strategies. These models can be broadly classified into chemically induced, autoimmune, genetic, and diet-induced systems, each reproducing specific aspects of disease pathophysiology while lacking full translational equivalence to human diabetes (67, 68).

Chemically induced models, particularly those based on streptozotocin (STZ), are widely used to generate insulin-deficient states through selective destruction of pancreatic β-cells. STZ is preferentially taken up via the GLUT2 transporter and induces DNA alkylation, activation of poly(ADP-ribose) polymerase (PARP), and depletion of intracellular NAD^+^, ultimately leading to β-cell necrosis and hyperglycemia (69). Depending on the dosing regimen, STZ can produce either rapid-onset diabetes or a more gradual phenotype with partial immune involvement (67). These models are highly reproducible and technically straightforward but do not recapitulate the autoimmune etiology of type 1 diabetes and may exhibit off-target toxicity.

Autoimmune diabetes is most comprehensively modeled in NOD mice, in which spontaneous disease develops due to T cell–mediated destruction of β-cells, accompanied by insulitis and autoantibody production (3, 70). This model captures key immunopathological features of human type 1 diabetes, including the involvement of both CD4^+^ and CD8^+^ T cells, but is limited by variability in disease onset and a pronounced sex bias (3).

Genetic models of type 2 diabetes frequently involve disruption of leptin signaling pathways. ob/ob mouse and db/db mouse exhibit hyperphagia, severe obesity, insulin resistance, and progressive hyperglycemia, making them valuable tools for studying metabolic dysregulation (71). However, these models have limited translational relevance, as monogenic defects in leptin signaling are rare in human diabetes. Additional genetic systems, such as Akita mice carrying mutations in the Ins2 gene, model β-cell dysfunction caused by endoplasmic reticulum stress and misfolded insulin accumulation, providing insight into non-immune mechanisms of β-cell failure (72).

Diet-induced obesity (DIO) models, typically generated through high-fat diet (HFD) feeding, reproduce key features of metabolic syndrome, including obesity, chronic low-grade inflammation, and peripheral insulin resistance (73, 74). These models involve disruption of insulin signaling pathways such as the IRS–PI3K–Akt axis and increased lipotoxic stress on β-cells. However, disease severity and progression depend strongly on genetic background and experimental conditions, and hyperglycemia may be less pronounced than in genetic models (73). To address this limitation, combined approaches such as HFD with low-dose STZ are frequently employed to model both insulin resistance and relative insulin deficiency (67).

It is important to mention that, the phenotype produced by each experimental model can also differ substantially between males and females. Sexual dimorphism may affect diabetes incidence, age of onset, hyperglycemia, insulin resistance, β-cell compensation, and susceptibility to β-cell injury. Moreover, the magnitude and direction of these differences are not only influenced by genetic background, but also by, age, hormonal status, diet composition, and experimental protocol. Therefore, sex should be considered not only as a reporting variable but also as a determinant of model phenotype and translational interpretation. The main features of these models are summarized in **Table 1**.

**Table 1:** Experimental mouse models of diabetes and their relevance for studies of insulin gene biology and translational diabetes research.

Model category	Model name/induction method	Primary mechanism	Key features	Strengths	Limitations	Implications for insulin gene biology and translational research	Human disease best represented	Sex differences/sexual dimorphism
Chemically induced	Streptozotocin (STZ)	Selective β-cell uptake via GLUT2 → DNA alkylation → PARP activation → NAD^+^ depletion → β-cell necrosis	Rapid or gradual hyperglycemia depending on dose; reproducible	Technically simple; highly reproducible; flexible dosing regimens	No autoimmune component; potential off-target toxicity; does not model T1D etiology	Allows study of insulin deficiency without confounding immune destruction; useful for testing insulin replacement or β-cell regeneration strategies	β-cell destruction (not autoimmune)	Sex effect depends on strain, age, dose, number of injections, and hormonal status. Females can show relative resistance to β-cell toxicity under some STZ regimens, but the direction and magnitude are protocol-dependent.
Autoimmune	NOD (Non-Obese Diabetic)	Spontaneous T-cell-mediated (CD4^+^ and CD8^+^) destruction of β-cells; insulitis; autoantibody production	Variable disease onset; sex bias (females > males); genetic complexity	Closest model to human T1D; captures key immunopathological features	Variable penetrance; high cost; sex bias; incomplete translation to human T1D	Central for studying insulin epitopes (e.g., Ins1 vs. Ins2 immunodominance); thymic expression differences matter	Type 1 diabetes	Strong female predominance in diabetes incidence in NOD mice (almost two folds higher); females generally develop insulitis and diabetes more frequently and/or earlier than males, although this bias varies with strain and housing conditions.
Genetic – leptin pathway	ob/ob (leptin deficient), db/db (leptin receptor deficient)	Monogenic disruption of leptin signaling → hyperphagia, severe obesity, insulin resistance, progressive hyperglycemia	Severe obesity; early onset; hyperinsulinemia followed by β-cell failure	Well-characterized; reproducible; models metabolic syndrome features	Monogenic leptin defects are very rare in human T2D; limited translational relevance for common T2D	Useful for studying insulin resistance and β-cell compensation; insulin gene expression changes occur secondary to metabolic stress	Severe obesity and insulin resistance (ob/ob); Advanced insulin-resistant diabetes (db/db)	Sex effects vary with genetic background and metabolic state. Female mice may display distinct phenotypes in obesity, insulin sensitivity, β-cell compensation, and diabetes progression; these effects differ across ob/ob and db/db strains (75).
Genetic – ER stress/insulin mutation	Akita (Ins2 C96Y)	Point mutation in Ins2 gene → misfolded proinsulin → ER stress → β-cell apoptosis	Autosomal dominant; progressive hyperglycemia; non-immune β-cell failure	Models monogenic diabetes (e.g., MODY10); ER stress mechanism relevant to both T1D and T2D	Ins1 remains intact; does not model autoimmune or polygenic T2D	Directly involves insulin gene mutation; allows study of proinsulin folding, ER stress, and sex differences (females protected)	Monogenic diabetes/MODY10	Male Akita mice generally develop more severe or earlier diabetes than females, consistent with greater metabolic demand and reduced β-cell resilience; sex differences can be modified by age and hormonal status (72).
Diet-induced	High-fat diet (HFD)	Chronic high-fat feeding → obesity, lipotoxicity, low-grade inflammation → IRS-PI3K-Akt disruption	Gradual onset; body weight increase; moderate hyperglycemia; insulin resistance	Models lifestyle-induced T2D; adjustable diet composition; no genetic manipulation required	Variable severity dependent on strain/duration; hyperglycemia often mild; requires prolonged feeding	Helps study insulin gene regulation under metabolic stress; can combine with STZ to induce insulin deficiency	Lifestyle-induced prediabetes/T2D	Females are often more resistant to HFD-induced weight gain, insulin resistance, and hyperglycemia than males, although the phenotype is strongly strain-, diet-, and duration-dependent (76).
Combined	HFD + low-dose STZ	HFD induces insulin resistance + low-dose STZ causes partial β-cell loss → relative insulin deficiency	More rapid and pronounced hyperglycemia than HFD alone; captures both insulin resistance and secretion defect	Better mimics late-stage T2D with overt hyperglycemia; shorter time course	Still lacks autoimmune component; two-hit protocol adds variability	Allows study of insulin gene expression in context of both peripheral resistance and β-cell dysfunction	Advanced type 2 diabetes	Combines sex-dependent responses to HFD and STZ. Females may require protocol adjustment to achieve comparable hyperglycemia; sex should therefore be treated as a design variable rather than a nuisance factor (77).

Overall, murine models of diabetes represent a spectrum of experimental systems that recapitulate discrete components of disease pathogenesis, including β-cell loss, immune-mediated destruction, and systemic metabolic dysfunction. While their combined use enhances mechanistic understanding, significant interspecies differences in immune regulation, glucose metabolism, and β-cell biology remain key limitations for translational application (68).

The evidence discussed in this section demonstrates that species-specific differences in insulin gene organization, regulation, and function substantially influence diabetes susceptibility and the interpretation of experimental models. While these evolutionary and molecular distinctions account for many discrepancies between murine and human studies, they do not fully explain the considerable heterogeneity observed in diabetes development and progression. Increasing evidence indicates that biological sex represents an additional layer of regulation that modifies β-cell biology, insulin sensitivity, immune responses, and disease susceptibility through both hormonal and chromosome-dependent mechanisms. Integrating species-specific biology with sex-dependent regulation is therefore essential for a more comprehensive understanding of diabetes pathophysiology and for improving the translational value of future experimental and clinical studies.

## 5 Gender differences in diabetes susceptibility

Sex, as biological difference between men and women, and gender, the socially constructed roles, behaviors, expressions, and norms that a society considers appropriate for men and women, are now becoming an interesting field of interest in studying non-transmitted diseases like T1D, T2D and obesity. So the main question became, how can sex related factors affect the risk of a person to develop T1D or T2D? Some of those factors are gender related like behavior, but most of them are sex related, such as sex chromosome, hormones, fat distribution, metabolism and glucose homeostasis, microflora, menopause and pregnancy. Statistically, men have shown more tendency to develop or be at risk of developing diabetes, in this section we will be discussing the main factors playing a role in these statistical findings.

### 5.1 Sex-dependent regulation of mouse Ins1 and Ins2

There is evidence that sex affects the two murine insulin genes unequally. The most direct β-cell transcriptomic evidence indicates that Ins2 is more consistently sex-biased than Ins1. In single-cell RNA-seq of mouse β cells, Liu et al. reported a significant male–female difference in Ins2 expression, supporting sexual dimorphism of the β-cell transcriptome; the same study also noted sex-associated differences in fasting insulin levels in healthy mice (78).

At the islet level, Jo et al. found no significant sex difference in Ins1 expression, but observed lower Ins2 expression in female islets, while also reporting sex differences in β-cell physiology and glucose homeostasis. This supports a model in which Ins2 is the more sex-sensitive transcript, at least under the tested physiological conditions (79).

Hormonal perturbation studies complicate the picture. In female rats exposed to dihydrotestosterone, Mishra et al. found increased mRNA levels of both Ins1 and Ins2, linking androgen excess to hyperinsulinemia through transcriptional regulation of insulin genes. However, the paper’s title and mechanistic emphasis focus particularly on increased Ins1 transcription, suggesting that androgens can regulate both genes but may preferentially amplify Ins1-driven insulin production in this model (80).

Estrogen studies mostly support a protective or adaptive role in β-cell physiology rather than a clean Ins1-versus-Ins2 regulatory distinction. Estradiol protects β cells from oxidative injury and diabetes in mice, improves insulin secretion under stress, and can increase Ins2 expression in some experimental settings, such as partial pancreatectomy models (81).

For autoimmune diabetes, the strongest sex-related evidence comes from NOD models with altered insulin gene dosage. Babaya et al. showed that male NOD Ins1+/−;Ins2−/− mice develop early diabetes, whereas female mice of the same genotype did not develop early diabetes by 10 weeks, despite reduced insulin content in both sexes. The authors linked this to insulin deficiency and male insulin resistance rather than to classical insulitis (82).

Overall, the most defensible conclusion is that sex does not appear to regulate Ins1 and Ins2 identically. Ins2 shows clearer sex-biased expression in β-cell transcriptomic datasets, whereas Ins1 may be more responsive in certain androgen-excess or insulin-demand states. In the thymus, the key tolerance-relevant gene is Ins2, but I did not find strong evidence that thymic Ins2 expression itself is directly regulated by sex in a simple male-versus-female manner. Instead, sex likely modifies disease through a combination of β-cell stress resilience, insulin demand, sex steroid signaling, and immune polarization.

### 5.2 Clinical and biological basis of sex differences in diabetes

Large epidemiological studies have established that diabetes prevalence and pathophysiology differ significantly between males and females (75). These differences extend beyond disease prevalence to include age at onset, metabolic phenotype, and the development of diabetes-associated complications.

In middle-aged populations, men generally have a higher prevalence of T2D than women (83), whereas pre-menopausal women have a lower rate of T2DM compared to both male and postmenopausal women (84). Research on hormone replacement therapy further supported this observation, demonstrating that fasting glucose levels increased significantly among women assigned to placebo but did not change among women receiving hormone therapy (85). During menopause, the decline in endogenous estrogen levels is associated with increased LDL-cholesterol, HbA1c, and a redistribution of body fat toward a more visceral pattern, all of which contribute to increased metabolic risk (86).

Sex differences are also evident in the development of chronic diabetic complications. In a 12-year follow-up study involving 1,053 individuals with T2D (453 men and 600 women), women exhibited higher incidences of diabetic kidney disease (36.1% vs. 31.4%) and peripheral artery disease (35.4% vs. 23.9%), whereas men more frequently developed diabetic retinopathy (37.0% vs. 27.3%) and coronary artery disease (36.2% vs. 34.5%). The incidence of peripheral neuropathy was comparable between the sexes (63.0% in men vs. 62.7% in women). After adjustment for potential confounding variables, women remained at significantly higher risk of peripheral artery disease (RR = 1.62; 95% CI, 1.20–2.17), but had lower risks of diabetic retinopathy (RR = 0.75; 95% CI, 0.59–0.97) and coronary artery disease (RR = 0.60; 95% CI, 0.49–0.75) than men. Collectively, these findings indicate that biological sex influences not only diabetes susceptibility but also the pattern of long-term vascular complications (87).

Notably, T1D represents an exception among autoimmune diseases, showing a male predominance after puberty (88, 89). One of the principal mechanisms underlying this observation involves central immune tolerance to insulin. The INS VNTR minisatellite on chromosome 11p15.5 (historically designated IDDM2) controls thymic insulin expression in medullary thymic epithelial cells. Individuals carrying class III alleles (140–210 repeats) express approximately two- to threefold higher levels of thymic INS mRNA than those carrying class I alleles (26–63 repeats), thereby facilitating more efficient negative selection of autoreactive T cells (64). This process requires AIRE-mediated chromatin remodeling at the VNTR locus through its PHD zinc-finger and LXXLL motifs, allowing transcription of tissue-restricted antigens within the thymus. Importantly, AIRE abundance, rather than VNTR class alone, is considered the primary determinant of inter-individual variability in thymic insulin expression.

Biological sex further modulates this process. Females express significantly less AIRE mRNA and protein in thymic epithelial cells after puberty, largely due to estrogen-driven epigenetic modifications at the AIRE promoter (90). This reduction may decrease the efficiency of central immune tolerance and contribute to sex-dependent differences in autoimmune susceptibility. Conversely, estrogen promotes the expansion and stabilization of FoxP3+ regulatory T cells through estrogen-response elements in the FOXP3 locus, illustrating that estrogen exerts both immune-stimulatory and immune-regulatory effects depending on the cellular context.

Beyond hormonal mechanisms, sex chromosome complement also contributes to immune dimorphism through X-linked gene dosage effects. Escape from X-chromosome inactivation, mediated by XIST, permits increased expression of immune-related genes including TLR7, FOXP3, CD40L and CXCR3, providing an additional mechanism through which biological sex influences autoimmune responses in diabetes (91).

This indicates that sex-dependent immune regulation is governed by the interaction of genetic susceptibility, thymic tolerance, hormonal signaling, and sex chromosome complement rather than by any single mechanism alone.

Experimental animal models provide mechanistic evidence supporting these clinical observations. Ovariectomized C57BL/6J mice develop decreased endogenous estrogen levels, impaired glycemic control, and reduced glucose tolerance. Importantly, this phenotype is largely reversed following estradiol treatment, demonstrating a direct protective role of estrogen in glucose homeostasis (92).

Lack of endogenous estrogens is associated with reduced expression of Syntaxin-1A (Synt-1A), a key component of the SNARE complex required for insulin granule exocytosis, together with increased hepatic expression of insulin-degrading enzyme (IDE), thereby reducing circulating insulin availability (93).

In addition to its effects on pancreatic β-cells, estrogen also regulates systemic metabolism. ERα activation in hepatocytes promotes fibroblast growth factor 21 (FGF21) synthesis, increasing energy expenditure and protecting against hepatic steatosis, adipose tissue accumulation, and insulin resistance (94). Consistent with these findings, female mice fed a high-fat diet retain partial protection against metabolic dysfunction through ERα-dependent signaling pathways, highlighting the central role of estrogen signaling in maintaining whole-body metabolic homeostasis (95).

In addition to endocrine and immunological mechanisms, intrinsic metabolic differences between males and females contribute to sex-specific diabetes susceptibility. Women are approximately 40% more insulin sensitive than men, demonstrating greater whole-body insulin sensitivity, enhanced skeletal muscle glucose uptake, a higher proportion of oxidative type I muscle fibers, increased capillary density, and differences in lipid metabolism. These characteristics improve metabolic flexibility and may partially explain the delayed onset of T2D observed in premenopausal women (75, 96). During prolonged fasting, females preferentially maintain hepatic lipid synthesis through amino acid metabolism, whereas males rely more heavily on carbohydrate-derived substrates. Furthermore, accumulating evidence suggests that male and female tissues differ in their susceptibility to oxidative stress, apoptosis, ferroptosis, and other forms of regulated cell death, potentially contributing to sex-specific differences in β-cell survival and disease progression (97).

### 5.3 Sex hormones regulate β-cell function and survival

Sex hormones, particularly estrogens, play a central role in maintaining pancreatic β-cell function and survival under both physiological and pathological conditions. Beyond regulating insulin secretion, estrogens protect β-cells against oxidative stress, endoplasmic reticulum stress, mitochondrial dysfunction, and multiple forms of regulated cell death.

Maturity-onset diabetes of the young type 10 (MODY10) is a monogenic form of diabetes caused by mutations in the INS gene that result in proinsulin misfolding and progressive β-cell dysfunction. Misfolded proinsulin triggers ER stress, activating the unfolded protein response (UPR), causing eventual β-cell death (98). Although apoptosis has long been considered the principal mechanism responsible for β-cell loss in MODY10, accumulating evidence suggests that additional forms of regulated cell death also contribute to disease progression. Oxidative stress and iron accumulation (two defining hallmarks of ferroptosis) have been increasingly recognized as important contributors to β-cell death (99). Mediators of islet oxidative stress, including glutathione peroxidase-4 (GPX4), have been identified as key endogenous inhibitors of ferroptosis, making the GPX4 pathway essential for maintaining β-cell viability.

GPX4 utilizes reduced glutathione (GSH) as a cofactor to convert toxic phospholipid hydroperoxides (PLOOH) into non-toxic phospholipid alcohols (PLOH), thereby preventing lipid peroxidation and ferroptotic cell death. Estrogen further enhances these protective mechanisms by promoting membrane lipid remodeling through activation of membrane-bound O-acyltransferase domain-containing proteins 1 and 2 (MBOAT1/2), increasing cellular resistance to lipid peroxidation (100, 101). Because estrogen signaling enhances resistance to lipid peroxidation, female β-cells are thought to possess a greater capacity to counteract ferroptotic stress than male β-cells, providing a potential mechanistic explanation for the reduced β-cell loss observed in several experimental models of diabetes.

Assessment of GPx4 expression in different pancreatic islet cell types revealed a predominant expression in β-cells (102). The remarkably high expression of GPX4 in β-cells, compared with other endocrine cell types, underscores their dependence on this antioxidant pathway and suggests that impairment of GPX4 activity renders β-cells particularly susceptible to the accumulation of toxic lipid peroxides and ferroptotic cell death (103). In fact, pancreatic islets are highly susceptible to ferroptosis-inducing conditions during islet isolation and transplantation. Pretreatment with ferroptosis inhibitors such as ferrostatin-1 or desferrioxamine (DFO) markedly reduces β-cell death, further supporting ferroptosis as a therapeutically relevant mechanism of β-cell loss (103).

A substantial body of evidence indicates that estrogen plays a protective role in maintaining glucose homeostasis. Studies using streptozotocin-induced diabetic mouse models demonstrate that female mice maintain better glucose tolerance and pancreatic function compared to males, and this protection is lost following ovariectomy. Male and ovariectomized female mice exhibit significantly higher fasting blood glucose, impaired glucose tolerance, and more severe pancreatic islet atrophy. Restoration of estrogen signaling through estradiol replacement largely reverses these metabolic abnormalities, demonstrating that endogenous estrogens preserve β-cell function and limit diabetes progression (104). Collectively, these findings identify estrogen as a critical determinant of pancreatic resilience under diabetic conditions.

Additional evidence for sex-dependent regulation of diabetes is provided by the Akita mouse model, in which a mutation in the Ins2 gene causes proinsulin misfolding, chronic ER stress, and progressive β-cell failure. In the Akita mouse model of type 1 diabetes, male mice exhibit more rapid progression of diabetes and more pronounced hyperphagia compared to females, a difference linked to distinct hypothalamic neuropeptide regulation. In males, hyperphagia is associated with reduced expression of the anorexigenic peptide proopiomelanocortin (POMC) and increased expression of the orexigenic neuropeptide Y (NPY), along with suppressed serum leptin levels. Castration normalized food intake, blood glucose, and leptin levels, and restored POMC and NPY expression, indicating a testosterone-dependent mechanism involving the leptin-POMC-NPY axis. In contrast, although female Akita mice also develop hyperphagia, they show no significant changes in POMC, NPY, or leptin levels. Instead, hyperphagia in females is associated with decreased expression of the anorexigenic peptides cocaine- and amphetamine-regulated transcript (CART) and corticotrophin-releasing factor (CRF), and ovariectomy has no effect on food intake or blood glucose. These findings demonstrate that the neuroendocrine regulation of energy balance differs fundamentally between the sexes during diabetes progression. Whereas males primarily rely on the leptin–POMC–NPY pathway, females appear to compensate through CART- and CRF-dependent mechanisms, highlighting sexually dimorphic adaptations of the hypothalamus to chronic insulin deficiency (105).

The protective effects of estrogen are mediated primarily through estrogen receptor α (ERα), although estrogen receptor β (ERβ) and the G protein-coupled estrogen receptor (GPER) also contribute to β-cell protection and metabolic regulation (S. 106). These receptors are expressed in multiple metabolic tissues, including pancreatic beta cells, liver, skeletal muscle, and adipose tissue. Activation of these receptors enhances insulin secretion, improves insulin sensitivity, preserves mitochondrial function, and protects β-cells from ER stress, oxidative stress, apoptosis, and ferroptosis. In pancreatic β-cells, ERα represents the predominant mediator of estrogen-induced cytoprotection, promoting insulin biosynthesis and secretion while limiting oxidative damage and preserving mitochondrial integrity.

In mice with specific myocyte ERα deletion, muscle-associated oxidative metabolism was altered and hyperglycaemia developed, indicating that estrogen signaling is essential for maintaining skeletal muscle insulin sensitivity and mitochondrial function (107). ERα activation maintains the functional integrity of the mitochondrial electron transport chain, thereby sustaining the oxidative capacity required for insulin-stimulated glucose uptake.

Within adipose tissue, ERα signaling promotes metabolic flexibility through AMPK- and ATGL-mediated lipolysis, increasing non-esterified fatty acid (NEFA) availability and stimulating UCP-1-dependent thermogenesis in brown adipose tissue (108). Brown adipose tissue is generally better preserved and more metabolically active in women than in men, contributing to greater energy expenditure (109). In addition, estrogen signaling suppresses adipose tissue inflammation and fibrosis, both of which are major drivers of insulin resistance (110). Following menopause, declining estrogen levels promote a shift from subcutaneous to visceral fat accumulation, thereby enhancing pro-inflammatory signaling and increasing metabolic risk (86).

In the liver, ERα regulates lipid synthesis, lipoprotein production, insulin-like growth factor-1 (IGF-1) expression, and the transcription of genes involved in gluconeogenesis and lipid metabolism, thereby contributing to systemic glucose homeostasis (111). Furthermore, estrogen promotes preferential subcutaneous fat deposition while limiting visceral and ectopic lipid accumulation, a body fat distribution that contributes to the greater insulin sensitivity observed in premenopausal women (112). These tissue-specific actions demonstrate that estrogen protects against diabetes not only by preserving pancreatic β-cell viability but also by coordinating glucose and lipid metabolism across multiple insulin-responsive organs.

The sex-dependent effects described above intersect with intracellular Ca2+ and ER homeostasis. Estrogen signaling can support β-cell resilience not only by improving systemic insulin sensitivity but also by limiting ER stress and preserving mitochondrial function. Conversely, loss of estrogen signaling can increase metabolic and cellular stress, potentially reducing the capacity of β-cells to maintain Ca2+ gradients, proinsulin folding, and secretory-granule homeostasis. These mechanisms provide a plausible cellular link between sex hormones and the differences in β-cell survival observed in STZ, Akita, and other diabetes models.

Importantly, the relationship is bidirectional: chronic ER stress and disturbed Ca2+ homeostasis can themselves impair insulin biosynthesis and secretion, activate calpain- and UPR-associated cell-death pathways, and reduce β-cell functional reserve. WFS1 and CISD2 therefore provide useful mechanistic models for understanding how ER Ca2+, mitochondrial Ca2+ transfer, proteostasis, and β-cell death converge with the sex-dependent pathways discussed in this section.

### 5.4 Sex differences in human islet function

While epidemiological studies demonstrate clear sex differences in diabetes susceptibility, understanding how these differences arise requires direct examination of pancreatic islet function. Animal models and high-resolution transcriptomic analyses of human donor cells have revealed that male and female islets differ not only in hormone responsiveness but also in gene expression, mitochondrial metabolism, and β-cell resilience under diabetic stress.

Sex differences have been observed in the onset of T2D, where men are more likely to develop the disease than women during early and middle adulthood. These clinical observations suggest that intrinsic differences in pancreatic islet biology may contribute to sex-specific diabetes susceptibility. Similar findings have been reported in the Zucker Fatty Diabetes Mellitus (ZFDM) rat model, in which male animals are markedly more susceptible to developing early-onset T2D. However, the ZFDM phenotype should not be interpreted as a direct model of the mechanisms responsible for sex differences in common human T2D. ZFDM rats carry a homozygous recessive mutation in the leptin receptor (fa/fa), producing a genetically defined metabolic context characterized by severe obesity, insulin resistance, and progressive β-cell dysfunction. Consequently, the magnitude and timing of the sex difference observed in this model are shaped by leptin-receptor deficiency and its downstream metabolic consequences and may not be generalizable to polygenic human T2D. The model is therefore most appropriately viewed as evidence that biological sex can modify β-cell adaptation and metabolic disease progression under a defined genetic background, rather than as evidence for a universal mechanism of sex-dependent diabetes susceptibility. In this context, ZFDM findings are most informative when considered together with human epidemiological studies and human islet transcriptomic and functional data (113).

To investigate the mechanisms underlying these sex differences, Dr. Norihide Yokoi and colleagues from the Kyoto University in Japan, examined the effects of sex hormones on the development of T2D and gene expression in the pancreatic islets in 6-week-old ZFDM rats homozygous for the leptin receptor mutation (fa/fa). Surgical removal of male gonads (orchiectomy) did not prevent the development of diabetes, only slightly lowering non-fasting blood glucose levels, suggesting that testicular androgens are not the primary drivers of male susceptibility in this model. In contrast, ovariectomy impaired glucose tolerance and accelerated pancreatic islet fibrosis and β-cell loss, despite causing little change in non-fasting blood glucose levels. These findings indicate that endogenous ovarian hormones play a critical role in preserving islet architecture and β-cell function. Interestingly, pair-feeding experiments produced a similar protective effect, indicating that reduced caloric intake contributes substantially to the metabolic benefits of E2 treatment. Moreover, direct E2 treatment of isolated male islets upregulated genes involved in insulin secretion (e.g., Abcc8, Cacna1d, Glp1r) and downregulated cell-cycle genes, pointing to cell-autonomous effects of estrogen on β-cell function and proliferation (114).

To further investigate the molecular basis of sex differences in T2D, researchers analyzed the GSE86473 dataset from the Gene Expression Omnibus (GEO). Differential expression analysis identified substantially more differentially expressed genes (DEGs) in females (237 genes) than in males (51 genes), indicating greater transcriptional remodeling during diabetes progression. Pathway enrichment analysis further demonstrated that males predominantly exhibited immune- and inflammation-related signatures, whereas females showed greater alterations in pancreatic metabolic and secretory pathways. Although several transcription factors (ATF1, IRF1, MAZ, SSRP1, TFDP1, and MXD3) and microRNAs were shared between the sexes, distinct hub genes and signaling pathways characterized each group (**Table 2**). Together, these findings suggest that male and female pancreatic tissues activate different molecular programs during T2D progression. Researchers analyzed the GSE86473 dataset from the Gene Expression Omnibus (GEO), to further investigate the molecular basis of sex differences in T2D and support investigation of sex-informed biomarkers and therapeutic strategies. Differential expression analysis identified substantially more differentially expressed genes (DEGs) in females (237 genes) than in males (51 genes), indicating greater transcriptional remodeling during diabetes progression. Pathway enrichment analysis further demonstrated that males predominantly exhibited immune- and inflammation-related signatures, whereas females showed greater alterations in pancreatic metabolic and secretory pathways. Although several transcription factors (ATF1, IRF1, MAZ, SSRP1, TFDP1, and MXD3) and microRNAs were shared between the sexes, distinct hub genes and signaling pathways characterized each group (**Table 2**). Together, these findings suggest that male and female pancreatic tissues activate different molecular programs during T2D progression, supporting further investigation of sex-informed biomarkers and therapeutic strategies.

**Table 2:** Summary of sex-specific transcriptomic alterations identified in the GSE86473 dataset from patients with type 2 diabetes.

Feature	Male T2D	Female T2D	Biological significance
Number of DEGs	51	237	Greater transcriptional remodeling occurs in females during T2D progression.
Predominant biological processes during T2D development	Immune activation, inflammation, antigen presentation	Pancreatic metabolism, digestive enzyme secretion, exocrine function	Indicates distinct molecular responses to diabetes in each sex.
Representative hub genes	LDHA, FLT1, IGFBP3, LILRB4, CSF2, IL1A, HLA-C, UBD, C1QB, HLA-DMA, HLA-DPB1, CFB, HLA-DRB1, HLA-A, CXCL8	CPA1, CPB1, CEL, PNLIP, CPA2, CELA3A, SYCN, PRSS3P2, CTRB1, AMY2A, CTRC, PNLIPRP1, PRSS1, HLA-DRB1, HLA-DQB1	Males exhibit stronger immune-related signatures, whereas females show greater alterations in pancreatic metabolic and secretory pathways.
Shared transcription factors	ATF1, IRF1, MAZ, SSRP1, TFDP1, MXD3	ATF1, IRF1, MAZ, SSRP1, TFDP1, MXD3	These common transcription factors likely represent conserved upstream regulators of islet gene expression, indicating that sex differences may arise through differential downstream target activation rather than entirely distinct transcriptional machinery.
Shared miRNAs	miR-124-3p, miR-107, miR-16-5p, miR-23b-3p, miR-27a-3p, miR-103a-3p, miR-155-5p, miR-1-3p	miR-124-3p, miR-107, miR-16-5p, miR-23b-3p, miR-27a-3p, miR-103a-3p, miR-155-5p, miR-1-3p	The presence of shared miRNAs suggests conserved post-transcriptional regulatory mechanisms in both sexes, while differences in their target genes may contribute to sex-specific molecular responses during T2D.
Clinical implication	Greater inflammatory signature	Greater metabolic remodeling	Supports further investigation of sex-informed biomarkers and therapeutic strategies for T2D.

However, interpretation of sex-specific transcriptomic remodeling in human islets requires consideration of substantial donor-level heterogeneity. Human pancreatic islet datasets are derived from individuals who differ not only in biological sex and diabetes status but also in age, body mass index (BMI), glycemic control, disease duration, race or ethnicity, medication exposure, tissue procurement conditions, and cellular composition. These variables can independently influence β-cell transcription, chromatin accessibility, mitochondrial function, inflammatory signaling, and endoplasmic reticulum stress. Accordingly, the greater autosomal transcriptional remodeling observed in female β-cells during T2D should be interpreted as an association rather than definitive evidence of a sex-specific causal mechanism. Although the 52-donor multimodal study incorporated donor-level matching and statistical approaches to reduce confounding, matching and adjustment cannot eliminate all residual confounding when sample sizes are limited or clinical metadata are incomplete. Disease duration and treatment exposure are particularly important because patients classified as having T2D may represent different stages of β-cell dysfunction and metabolic disease progression. Replication in larger, independently collected cohorts with detailed donor-level metadata and longitudinal clinical information will therefore be important to establish the robustness and causal relevance of these sex-associated molecular signatures.

To further characterize the cellular basis of sex-specific differences in diabetes, researchers integrated single-cell RNA sequencing (scRNA-seq), single-nucleus assay for transposase-accessible chromatin sequencing (snATAC-seq), hormone secretion assays, and bioenergetic analyses from human pancreatic islets obtained from 52 donors with and without T2D. In islets from nondiabetic (ND) donors, sex-dependent differences in chromatin accessibility and gene expression were largely restricted to sex chromosome-linked genes, particularly the X-linked KDM6A in females and the Y-linked KDM5D in males. Although sex chromosome-associated differences persisted in T2D islets, disease progression was accompanied by the emergence of extensive autosomal transcriptional differences between males and females, suggesting that diabetes is associated with enhanced sex-specific molecular remodeling beyond the differences predominantly observed on sex chromosomes in non-diabetic islets. Comparison of β-cells from T2D and ND donors further demonstrated that female β-cells underwent substantially greater autosomal transcriptional remodeling than male β-cells during disease progression. Gene set enrichment analysis revealed distinct pathogenic mechanisms in each sex. Female β-cells exhibited suppression of oxidative phosphorylation and electron transport chain pathways, consistent with mitochondrial dysfunction, whereas male β-cells showed predominant impairment of insulin secretion pathways. Thus, although sex-specific differences in gene accessibility and expression of cultured ND human islets predominantly affect sex chromosome genes, major differences in autosomal gene expression between sexes appear during the transition to T2D, with mitochondrial dysfunction particularly evident in female β-cells.

Independent analyses of publicly available human islet datasets, including the Human Pancreas Analysis Program (HPAP) and HumanIslets.com, further support the existence of sex-specific differences in pancreatic islet biology. Among non-diabetic donors, female islets contain a greater proportion of α-cells and exhibit enhanced expression of ribosomal and mitochondrial pathways in both α- and β-cells, despite displaying lower spare respiratory capacity than male islets. During T2D progression, however, male and female islets undergo distinct molecular and functional adaptations. Female islets preserve insulin content and β-cell function more effectively, whereas male islets exhibit more pronounced defects in insulin secretion. Collectively, evidence from animal models, bulk transcriptomic analyses, and single-cell multiomic studies demonstrates that biological sex influences pancreatic islet function at multiple levels, including cellular composition, gene regulation, mitochondrial metabolism, and insulin secretory capacity. These findings indicate that diabetes progresses through distinct molecular pathways in males and females and underscore the importance of incorporating sex as a biological variable in experimental studies and in the development of precision therapies for diabetes (115).

### 5.5 Mechanisms beyond sex hormones

The evidence reviewed above can be integrated into a multilevel framework in which sex hormones, sex chromosome dosage, tissue-specific metabolic remodeling, and intracellular β-cell resilience converge to determine diabetes susceptibility. **Figure 6** summarizes these interactions and highlights the connection between systemic sex-dependent regulation and β-cell-intrinsic mechanisms.

**Figure 6:**
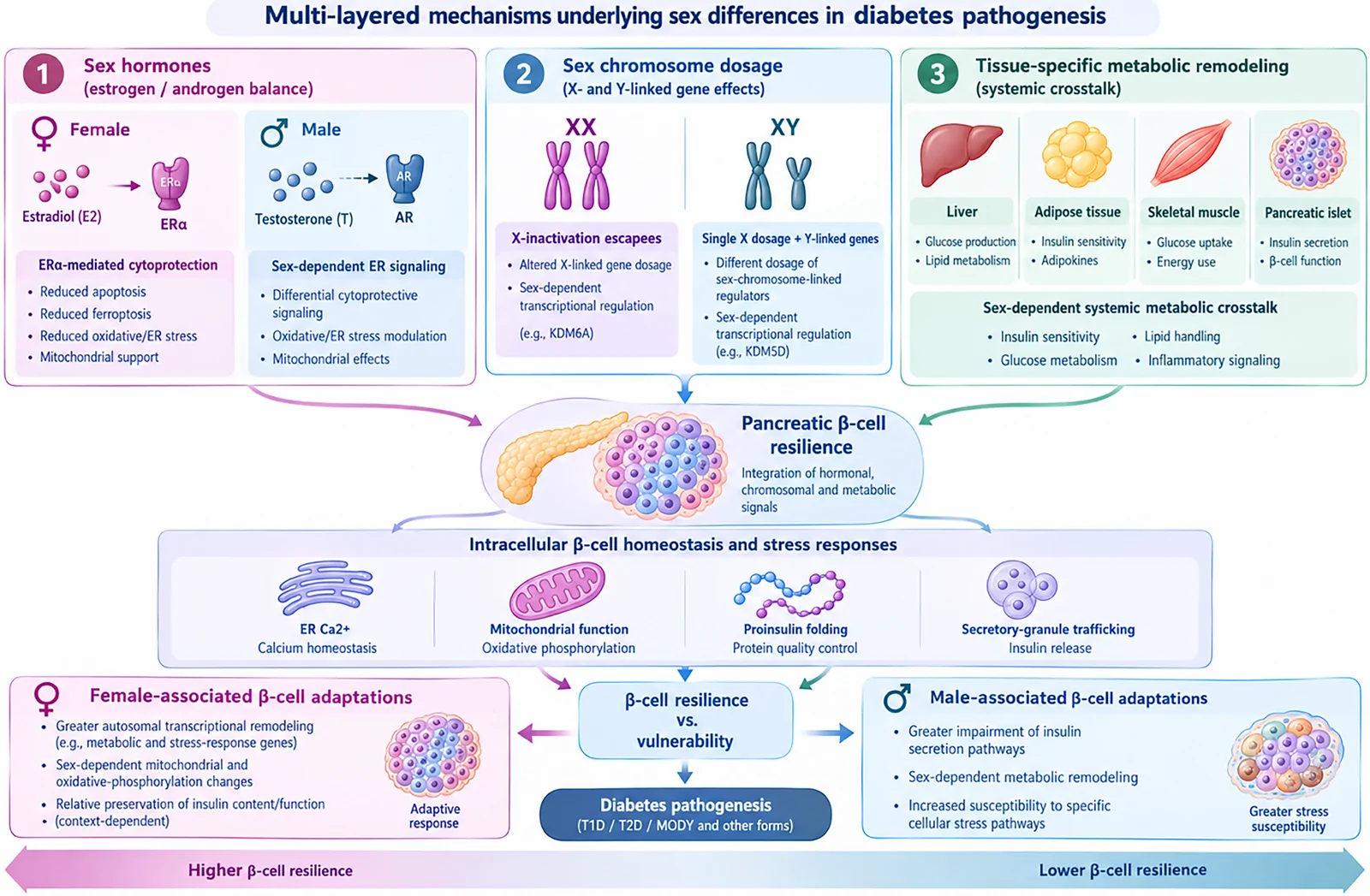
Integrative framework of sex differences in diabetes susceptibility. Sex hormones and ERα signaling, sex chromosome dosage and X-inactivation escapees, and tissue-specific metabolic remodeling converge on β-cell intracellular homeostasis, including ER Ca2+, mitochondrial function, proinsulin folding, and secretory-granule trafficking. These mechanisms collectively influence β-cell resilience and ultimately diabetes susceptibility and therapeutic response.

Although sex hormones, particularly estrogens, are major determinants of diabetes susceptibility, accumulating evidence indicates that sex differences also arise from hormone-independent mechanisms. Sex chromosome complement, epigenetic regulation, and gut microbiota composition independently influence metabolism, immune function, and pancreatic β-cell biology, thereby contributing to sex-specific susceptibility to both T1D and T2D.

Sex chromosomes are a crucial contributing factor to the sexual dimorphisms of adipose tissue distribution and the subsequent metabolic complications of obesity. Human sex chromosome anomalies such as Klinefelter syndrome (XXY) and Turner syndrome (XO) are a good example to analyze any changes in metabolism or immune system (116). In Klinefelter syndrome, the most common sex chromosome disorder in men, patients typically present with hypergonadotropic hypogonadism (HH), known as primary hypogonadism, a condition where the testes fail to produce sufficient testosterone, triggering the pituitary gland to secrete high levels of gonadotropins (FSH and LH) as a compensatory response. These changes in sex hormones level are often accompanied by an impairment of spermatogenesis, and the presence of a clinical syndrome mainly characterized by sexual dysfunction, generalized asthenia, reduction of testis volume, and gynecomastia, together with anemia and deterioration of muscle mass and strength and bone status (117). Moreover, hypogonadism is often associated with metabolic comorbidities, such as obesity, insulin resistance (IR), metabolic syndrome (MetS), and type-2 diabetes mellitus (T2-DM) (118–120).

Turner syndrome (TS) patients (the most common sex disorder observed in females, whereby one of the X chromosomes are partially or completely missing) have dramatically reduced gonadal hormone levels. These patients also lack protection against abdominal obesity and the relative risk of diabetes is elevenfold for T1DM and fourfold for T2DM, with onset near 36 years - about twenty years earlier than in the general population (121). Epidemiologically, it has been shown that impaired glucose tolerance affects 10%–78% of girls and women with TS, compared with ~5% of age-matched controls (122, 123). Importantly, subtle β-cell defects appear in childhood: up to 40% of girls with TS aged 5–12 years show asymptomatic hyperglycemia (124). Rigorous metabolic studies, including hyperinsulinemic-euglycemic clamps, the gold standard for insulin sensitivity, and oral glucose tolerance tests (OGTT), consistently demonstrate preserved or even enhanced insulin sensitivity and exaggerated glycemic excursions with blunted insulin responses, respectively, when matched for adiposity (125, 126) These findings strongly indicate that the primary defect in TS is not insulin resistance but an intrinsic, early-onset impairment of pancreatic β-cell secretion (127, 128).

Several experiments were conducted on genadectomized unique mouse models; gonadectomized male and female mice carrying XX chromosome complements in comparison to gonadectomized mice carrying the XY chromosome complements developed worse obesity disease outcomes (i.e. increased adiposity, increased satiety, and elevated lipid and insulin levels) (X. 129). In another model, gonadectomized mice carrying XO and XXY chromosome complements revealed that the differences between the XX and XY mice are due to the additional X chromosome (or “X chromosome dosage”) rather than the lack of a Y chromosome. Indeed, several genes that escape X chromosome inactivation are highly expressed in adipose and liver tissues, both of which are key regulators of metabolism. Thus, the X chromosome may be an important factor in addition to gonads/sex hormones that causes sex differences in obesity and metabolism (X. 129).

Notably, the presence of XX and XY chromosomes influence the developmental path between sexes and gonadal hormones. This ultimately affects the gene expression that may underpin the differences in obesity and metabolism observed between males and females. Although largely attributed to sex hormones and sex chromosomes, the sexual dimorphism of obesity has also been partially credited to sex differences in the microflora residing in our intestines.

A more accurate approach may be to detect obesity-related changes to the genus, family and species levels within the gut microbiome (130). Beneficial bacteria such as *Akkermansia muciniphila* and members of the Bifidobacterium genus have a negative correlation in the development of obesity (131, 132). The beneficial effects of *A. muciniphila* on the intestinal epithelial barrier have long been reported, as it a highly effective mucin-degrading bacterium, with the ability to use various enzyme combinations to hydrolyze up to 85% of mucin structures within the gut (133). A reduction in *A. muciniphila* is associated with increased intestinal permeability or a “leaky gut” – a hallmark of gut dysbiosis in obesity (134). Intestinal permeability allows leakage of water, proteins and other endotoxic molecules such as lipopolysaccharide (LPS) into systemic circulation with the ability to reach other organs and tissues (135). High circulating levels of LPS, termed metabolic endotoxemia, promotes further inflammation, weight gain and diabetes in experimental animals and humans. Recent studies have explored the possibility of using A. muciniphila-associated therapies as a next-generation treatment for obesity (136). Opposingly, harmful bacteria such as those from the Desulfovibrio, Fusobacterium and Bilophila genera are positively correlated with obesity (131, 132, 137).

(138, 139) In the male gut microbiota for example, *Veillonella* genera are found in higher abundance in children with type 1 diabetes. In the same time, *Roseburia genera* is found to improve metabolic alterations brought on by high fat diets (140). Likewise, for females, Bilophila genera aggravates metabolic dysfunction, however, Bacteroides genera has numerous metabolic benefits on the host (141, 142). Moreover, differences in the abundance of specific genera are also observed in metabolic syndrome patients. Higher abundances of *Veillonella*, *Methanobrevibacter*, *Acidaminococcus*, *Clostridium*, *Roseburia* and *Faecalibacterium* genera in males, whereas genera such as *Bilophila*, *Ruminococcus* and *Bacteroides* were greater in females (138, 139). These findings suggest that it is not simply the abundance of specific bacteria in the gut microbiota that determine health and disease within the host, but their metabolic activity, interaction with diet and the host.

The evidence reviewed in this section indicates that sex differences arise from multiple interacting layers of biological regulation rather than from the action of sex hormones alone. Differential insulin gene expression, hormone-dependent β-cell protection, sex-specific chromatin organization, and chromosome-driven transcriptional programs collectively influence glucose homeostasis and diabetes susceptibility. Importantly, these mechanisms interact with the species-specific differences discussed in earlier sections, emphasizing that both biological sex and species should be considered fundamental variables when designing experiments and interpreting diabetes research.

## 6 Implications for precision medicine

The growing recognition that diabetes develops through sex-specific molecular and physiological mechanisms has important implications for precision medicine. Incorporating biological sex into experimental design, clinical trials, and therapeutic development has the potential to improve disease prediction, treatment efficacy, and long-term outcomes. This section discusses how current knowledge of sex differences can be translated into more personalized approaches for diabetes research and patient care.

Despite increasing recognition of sex differences in diabetes, females remain underrepresented in both preclinical and clinical research. Male rodents and male-derived cell lines continue to dominate experimental studies because females are generally more resistant to diabetes induction in commonly used models, including high-fat diet, streptozotocin, and spontaneous models such as the Akita mouse. In addition, fluctuations in ovarian hormone levels are often perceived as a source of experimental variability, further discouraging the inclusion of females. Consequently, many mechanistic findings are generated and validated primarily in males, limiting their translational applicability to women (143, 144).

### 6.1 Sex-stratified clinical trial design

Sex-stratified clinical trial design intentionally balances, recruits and analyzes groups of participants in a clinical trial based on the sex, in order to differentiate between treatment efficiency, safety and outcomes. Usually, participants are divided into strata (male/female) before randomization, thus ensuring equal distribution within each sex.

Historically, cardiovascular and metabolic trials have either under-represented women or failed to prespecify sex specific analyses, leading to missed opportunities to detect differential treatment effects. For example, Large-scale meta-analyses have consistently shown that T2D confers a greater excess risk of macrovascular complications in women compared with men. The relative risk of coronary heart disease (CHD) is estimated to be 44% higher in women; the risk of stroke is 27% higher, the occlusive vascular mortality rate is nearly 50% higher, and the risk of vascular dementia is 19% higher (83, 145, 146). These disparities have been attributed to the interaction between sex and gender factors in the development, risk, and outcomes of diabetes (83).

Moreover, this challenge is not limited to clinical trials, but also to data analysis. A recent study using Framingham Heart Study data and genome wide association studies (GWAS) found that although nine T2DM-associated loci exhibit sex-dimorphic genetic effects (e.g., variants near CMIP, KLF14, and LYPLAL1), polygenic risk scores derived from sex-stratified GWAS did not outperform those from sex-combined analyses. The authors concluded that current sex-specific sample sizes are insufficient to improve predictive accuracy, highlighting the pressing need for larger sex-stratified studies(N. 147). For type 1 diabetes, immunomodulatory trials (e.g., anti-CD3, CTLA-4 Ig) have rarely reported outcomes by sex (148), despite clear evidence from the NOD mouse model that autoimmunity progresses differently in males and females (92). Prospective, adequately powered sex-disaggregated analyses are needed to determine when genuine sex-by-treatment interactions exist and whether they are clinically actionable. Regulatory and reporting frameworks increasingly support sex-disaggregated presentation of efficacy and safety outcomes, but implementation remains inconsistent (N. 147).

Beyond trial design, the growing understanding of estrogen’s protective effects opens the door to estrogen receptor (ER) agonists as potential adjuvant therapy for both type 1 and type 2 diabetes. In animal models, 17β-estradiol (E2) increases insulin sensitivity, protects beta cells, while lowering ER stress and suppressing ferroptosis (mechanisms are relevant to both types of diabetes) (81, 149, 150). On the other hand, selective ERα agonists (propyl pyrazole triol (PPT) and compound 17) showed the same effect on rodents without achieving the feminizing effect of natural estrogen therapy (151). In the clinical trials, these approaches are facing certain safety concerns, Women’s Health Initiative (WHI) has documented that unopposed ERα activation significantly raised the risk of endometrial cancer (152). Postmenopausal hormones have been initiated in menopausal women for the treatment of vasomotor symptoms, mood disturbances, vaginal dryness, and prevention of rapid bone loss for several decades. Despite a paucity of data on effects of initiating hormone use in older women, postmenopausal hormones have also been promoted for the prevention of CHD, osteoporotic fractures, and other diseases that occur years after menopause (153). It is generally recommended that women with a uterus be prescribed a combination of estrogen and progestin to prevent endometrial hyperplasia or cancer, whereas women with a hysterectomy receive unopposed estrogen. The purported benefits of estrogen are assumed to be similar for combined hormones, although relatively few studies have included long-term estrogen plus progestin users, particularly those taking continuous progestin. Reports of greater risk of breast cancer with cyclic estrogen/progestin combinations vs. unopposed estrogen (154, 155) highlight the need to determine the risks and benefits for both estrogen and combined hormones in appropriate clinical populations, including older women (152).

### 6.2 Sex differences in therapeutic response to antidiabetic drugs

Sex may influence pharmacokinetics (PK), pharmacodynamics (PD), efficacy, and adverse-effect profiles of antidiabetic therapies, but available evidence does not support a universal rule that men and women require different drugs or doses. Apparent sex differences can reflect body composition, renal function, disease severity, baseline cardiovascular or renal risk, hormonal status, concomitant medications, and differences in trial representation. The most appropriate interpretation is therefore drug- and outcome-specific, with adequately powered sex-disaggregated analyses needed to distinguish true sex-by-treatment interactions from confounding.

GLP-1 receptor agonists (GLP-1RAs) provide evidence for a quantitative, rather than necessarily qualitative, sex difference in treatment response. A 2025 systematic review and meta-analysis of 14 studies found that women achieved greater weight reduction than men with GLP-1RAs, with the difference observed across several agents and becoming more apparent with greater absolute weight loss (156). Potential explanations include differences in drug exposure and clearance, body composition, and interactions between GLP-1 signaling and sex hormones. For example, sex-disaggregated pharmacokinetic analyses of liraglutide have reported higher exposure in women at comparable body weight. Importantly, greater weight loss in women does not imply greater overall cardiovascular benefit: pooled cardiovascular and renal outcome trials indicate that GLP-1RAs reduce major outcomes in both sexes, without convincing evidence of a clinically meaningful sex-by-treatment interaction (157, 158).

For sodium-glucose cotransporter 2 inhibitors (SGLT2is), sex-specific PK/PD evidence remains limited and somewhat inconsistent. Some pharmacokinetic analyses suggest higher systemic exposure in women for individual agents, whereas other analyses have not identified differences considered clinically important. More robust clinical evidence indicates that the major cardiovascular and kidney benefits of SGLT2is are broadly preserved in both sexes. A 2026 meta-analysis of 25 cardiovascular outcome trials including more than 185,000 participants found no significant heterogeneity in cardiovascular or renal efficacy between sexes treatment with GLP-1RAs or SGLT2is, although women represented only about one-third of participants (158). Thus, current evidence supports comparable effectiveness across sexes while simultaneously highlighting the need for better female representation and prospective sex-specific analyses.

Thiazolidinediones (TZDs) illustrate a more complex interaction between sex and metabolic phenotype. In routine clinical data and randomized trial datasets, sex and BMI jointly modified the relative glycemic response to TZDs versus sulfonylureas. Non-obese men showed a greater HbA1c reduction with sulfonylureas, whereas obese women showed greater glycemic benefit with TZDs. However, obese women also had greater risks of TZD-associated weight gain and edema, and fracture risk is an additional safety consideration. These findings suggest that sex may become clinically informative when interpreted together with body composition and baseline metabolic phenotype rather than as an isolated determinant (159).

For metformin, evidence for sex-dependent efficacy or PK differences is substantially weaker. A recent review concluded that antihyperglycemic efficacy and PK are generally not clearly different between women and men when body weight and renal function are considered, whereas adverse drug reactions may be reported more frequently in women (160). This distinction is important because higher reporting of adverse events does not by itself demonstrate a pharmacological sex difference; differences in comorbidity, prescribing patterns, exposure, and healthcare utilization may contribute.

Overall, the strongest current quantitative evidence for sex differences among these drug classes concerns some GLP-1RA weight-loss outcomes and the sex-by-BMI interaction observed with TZDs. In contrast, the major cardiovascular and renal benefits of GLP-1RAs and SGLT2is appear broadly comparable between sexes, while metformin shows limited evidence for clinically meaningful sex-dependent efficacy. These findings support a precision-medicine framework based on sex-aware evaluation of PK, PD, efficacy, and safety rather than routine sex-specific prescribing. Future trials should prespecify sex-by-treatment interaction analyses, report absolute as well as relative treatment effects by sex, and ensure adequate representation of women and men.

Despite the fact that progestin co-administration can provide uterine protection, it frequently compromises metabolic health by inducing insulin resistance. Researchers are trying to overcome this problem by developing Tissue Selective Estrogen Complexes (TSECs) and Estrogen conjugated ligands. These tools aim to selectively target organs that regulate metabolism (liver and pancreas) while avoiding uterine stimulation and systemic toxicity.

## 7 Conclusions and future directions

Decades of research have identified the insulin gene as one of the most well-studied loci in endocrinology. However, recent advances in genomics, epigenomics, and single-cell technologies have shown that insulin biology is much more complex than previously thought. While insulin plays a highly conserved role across mammals, significant differences in gene structure, regulatory mechanisms, genomic environments, and pancreatic islet biology set humans apart from the experimental models that underpin much of current understanding. In mice, the insulin system includes two non-allelic genes (*Ins1* and *Ins2*), which are more an evolutionary adaptation than mere duplication. These differences impact β-cell function, immune tolerance, and diabetes risk, and do not have direct equivalents in humans. Therefore, it is crucial to interpret findings from mouse models within their specific biological context instead of assuming they directly mirror human disease.

Biological sex introduces an additional complexity that interacts with, rather than simply complements, species-specific biology. This review presents evidence from epidemiological studies, experimental models, transcriptomic analyses, and single-cell multi-omics, consistently showing that males and females differ in β-cell function, insulin secretion, mitochondrial metabolism, immune regulation, and vulnerability to both type 1 and type 2 diabetes. Crucially, these differences cannot be fully explained by circulating sex hormones alone. They result from a complex interplay of hormonal signaling, sex chromosome complement, epigenetic regulation, developmental programming, and environmental factors. Notably, many sex-dependent molecular signatures become more pronounced during diabetes progression, indicating that biological sex influences not only disease risk but also the mechanisms driving diabetes development.

Instead of undervaluing rodent models, these insights clarify their appropriate use. The key issue is no longer whether mice accurately represent human diabetes, but which questions they are best suited to explore and where their limitations matter biologically. Similarly, routinely excluding one sex or combining male and female data without acknowledging sex-specific responses can hide mechanisms that influence therapeutic success or disease progression in humans. Therefore, considering sex as a fundamental biological variable should be seen not as an extra burden but as a vital part of conducting rigorous, reproducible diabetes research.

Emerging technologies such as single-cell multi-omics, spatial transcriptomics, genome editing, and stem cell-derived human islet models are starting to close the gap between experimental systems and human disease. These methods offer new opportunities to analyze species-specific regulatory networks and explore how biological sex influences β-cell function and diabetes risk at the cellular level. However, obstacles such as limited access to high-quality human tissue, donor variability, and the need for larger, well-powered studies continue to hinder a comprehensive understanding of sex-by-species interactions.

The future of diabetes research depends not on replacing existing animal models but on combining comparative genomics, human tissue analysis, and sex-specific biological data into a cohesive experimental approach. This strategy will enhance the interpretation of preclinical results, increase their translational utility, and accelerate the development of personalized medicine approaches that account for biological diversity among patients. Viewing species differences and biological sex as key factors influencing insulin biology rather than as sources of variability is a crucial step toward more reproducible, mechanistically insightful, and clinically relevant diabetes research.
